# The Contribution of Individual Exercise Training Components to Clinical Outcomes in Randomised Controlled Trials of Cardiac Rehabilitation: A Systematic Review and Meta-regression

**DOI:** 10.1186/s40798-017-0086-z

**Published:** 2017-05-05

**Authors:** Bridget Abell, Paul Glasziou, Tammy Hoffmann

**Affiliations:** Centre for Research in Evidence-Based Practice, Faculty of Health Sciences and Medicine, Bond University, Gold Coast, Queensland 4229 Australia

## Abstract

**Background:**

While the clinical benefits of exercise-based cardiac rehabilitation are well established, there is extensive variation in the interventions used within these trials. It is unknown whether variations in individual components of these exercise interventions provide different relative contributions to overall clinical outcomes. This study aims to systematically examine the relationship between individual components of the exercise intervention in cardiac rehabilitation (such as intensity and frequency) and clinical outcomes for people with coronary heart disease.

**Methods:**

In this systematic review, eligible trials were identified via searches of databases (PubMed, Allied and Complementary Medicine, EMBASE, PEDro, Science Citation Index Expanded, CINAHL, The Cochrane Library, SPORTDiscus) from citation tracking and hand-searching. Studies were included if they were randomised trials of a structured exercise intervention (versus usual care) for participants with coronary heart disease and reported at least one of cardiovascular mortality, total mortality, myocardial infarction or revascularisation outcomes. Each included trial was assessed using the Cochrane Risk of Bias Tool. Authors were also contacted for missing intervention details or data. Random effects meta-analysis was performed to calculate a summary risk ratio (RR) with 95% confidence interval (CI) for the effect of exercise on outcomes. Random effects meta-regression and subgroup analyses were conducted to examine the association between pre-specified co-variates (exercise components or trial characteristics) and each clinical outcome.

**Results:**

Sixty-nine trials were included, evaluating 72 interventions which differed markedly in terms of exercise components. Exercise-based cardiac rehabilitation was effective in reducing cardiovascular mortality (RR 0.74, 95% CI 0.65 to 0.86), total mortality (RR 0.90, 95% CI 0.83 to 0.99) and myocardial infarction (RR 0.80, 95% CI 0.70 to 0.92). This effect generally demonstrated no significant differences across subgroups of patients who received various types of usual care, more or less than 150 min of exercise per week and of differing cardiac aetiologies. There was however some heterogeneity observed in the efficacy of cardiac rehabilitation in reducing total mortality based on the presence of lipid lowering therapy (*I* ^2^ = 48%, *p* = 0.15 for subgroup treatment interaction effect). No single exercise component was identified through meta-regression as a significant predictor of mortality outcomes, although reductions in both total (RR 0.81, *p* = 0.042) and cardiovascular mortality (RR 0.72, *p* = 0.045) were observed in trials which reported high levels of participant exercise adherence, versus those which reported lower levels. A dose-response relationship was found between an increasing exercise session time and increasing risk of myocardial infarction (RR 1.01, *p* = 0.011) and the highest intensity of exercise prescribed and an increasing risk of percutaneous coronary intervention (RR 1.05, *p* = 0.047).

**Conclusions:**

Exercise-based cardiac rehabilitation is effective at reducing important clinical outcomes in patients with coronary heart disease. While our analysis was constrained by the quality of included trials and missing information about intervention components, there appears to be little differential effect of variations in exercise intervention, particularly on mortality outcomes. Given the observed effect between higher adherence and improved outcomes, it may be more important to provide exercise-based cardiac rehabilitation programs which focus on achieving increased adherence to the exercise intervention.

**Electronic supplementary material:**

The online version of this article (doi:10.1186/s40798-017-0086-z) contains supplementary material, which is available to authorized users.

## Key Points

- Exercise-based cardiac rehabilitation interventions demonstrate considerable heterogeneity in format, yet few individual exercise training components predict better or worse clinical outcomes.
- Adherence to the exercise intervention as prescribed may however be important in affecting mortality outcomes.
- Clinicians should be aware that structured exercise programs can be flexible in design, without greatly impacting on the clinical outcomes expected.

## Background

While ongoing improvements in diagnosis and treatment have resulted in a steady increase in survival rates from major coronary events [1, 2], the burden of coronary heart disease on public health remains a substantial problem. With an increasing number of patients surviving acute cardiac events, the impetus to use effective secondary prevention strategies grows. However, this need is not being met, with up to 40% of all coronary events occurring in patients who have previously been diagnosed or hospitalised with the disease [3–5], and well-documented evidence-practice gaps in the use of effective therapies [6, 7]. Reducing the frequency of these recurrent events by enhancing the uptake of effective pharmacological and non-pharmacological interventions should therefore be considered an important health care priority.

While the benefits of exercise-based cardiac rehabilitation in the secondary prevention of coronary heart disease are well established [8–10], the complex nature of this intervention presents a substantial challenge to its implementation. Individual trial results vary considerably in terms of effectiveness, as well as in the type and ‘dose’ of exercise intervention provided, making it difficult to synthesise and translate these findings into practice in a way which provides optimal patient benefit. This problem has been compounded by incomplete reporting of intervention details in a substantial proportion of cardiac rehabilitation trials [11, 12]. In turn, this has hampered past attempts to understand how, and which, intervention characteristics relate to clinical outcomes.

Given the substantial variability of interventions in cardiac rehabilitation trials, it is pertinent to explore whether the differences in exercise interventions are contributing to the differences in observed effectiveness. While meta-analyses of these trials have provided evidence for the overall effectiveness of cardiac rehabilitation, vital content about the individual interventions and their effective components had been lost in the process of pooling these studies. An opportunity exists however to open this ‘black box’ of pooled interventions, by using meta-regression techniques to perform a more robust examination of the key intervention characteristics which may be associated with positive clinical outcomes [13, 14]. Past attempts to use this technique with cardiac rehabilitation have however only used a crude measure of exercise dose [8, 10, 15], excluded exercise interventions without a multi-faceted secondary prevention approach [16] or examined only intermediate outcomes such as cardiorespiratory fitness [17, 18].

The aim of this systematic review of randomised controlled trials of exercise-based cardiac rehabilitation for patients with coronary heart disease was to use meta-regression and subgroup meta-analysis to explore the contribution of individual exercise characteristics to clinical outcomes. This review expands on previous analyses by separating the exercise intervention into its smallest component parts, as well as obtaining and incorporating as many details as possible about previously unpublished intervention characteristics directly from trial authors.

## Methods

### Inclusion Criteria

Studies were eligible if they were randomised controlled trials with at least one arm that compared exercise-based cardiac rehabilitation to usual care, and which reported at least one of the following outcomes: total mortality, cardiovascular mortality, myocardial infarction, coronary artery bypass graft (CABG) or percutaneous coronary intervention (PCI). Trial participants could comprise men or women of any age who had been diagnosed with coronary heart disease, suffered a myocardial infarction, or undergone either CABG or PCI procedures.

Cardiac rehabilitation could have been provided in any setting (e.g. home, community or outpatient centre) but must have involved the prescription of a structured exercise program (either supervised or unsupervised), with or without the addition of lifestyle modification and counselling. Unsupervised home-based interventions were required to comprise a structured and detailed exercise prescription for participants (e.g. specific intensity, frequency and duration of individual sessions) with regular staff review, in a similar manner to centre-based programs. Including only structured exercise interventions (and excluding those which offered only general exercise advice, e.g. perform 150 min of exercise per week, walk daily) allowed for a more robust examination of the effect of specific exercise variables on outcomes.

Trials with a follow-up period of <3 months, with inadequate randomisation techniques, or those reporting on heart failure programs, were excluded. Trials published in abstract form only (from conference proceedings) were eligible for inclusion only if authors responded to email requests for further study information to determine if they met all eligibility criteria.

### Search Strategy and Selection of Studies

We conducted a structured search (last run on 28 January 2016) of the following databases: PubMed, Allied and Complementary Medicine, EMBASE, PEDro, Science Citation Index Expanded (via Web Of Science), CINAHL, The Cochrane Library and SPORTDiscus. The search strategy was developed in conjunction with an experienced medical librarian, consisted of a variety of exercise-based rehabilitation terms combined with coronary heart disease descriptors and used methodological filters to limit the results to randomised controlled trials, meta-analyses and systematic reviews. Variations of the following were searched, using a combination of text words and index terms: *coronary artery disease*, *ischemic heart disease*, *cardiovascular disease*, *myocardial infarction*, *angina*, *CABG*, *PTCA*, *rehabilitation*, *cardiac rehabilitation*, *exercise*, *physiotherapy*, *physical fitness*, *exercise training*, *training program*, *aerobic exercise*, *randomised controlled trial*, *random*, *controlled trial*, *meta analysis*, *systematic review* and *clinical trial* (Additional file 1: Appendix S1 contains full search strategy).

In order to identify further trials, the reference lists of all eligible studies were hand-searched, as well as those of previously published cardiac rehabilitation meta-analyses and systematic reviews. The process was supplemented further with searches of conference proceedings in the field from the previous 2 years (World Congress of Cardiology, American Heart Association Scientific Sessions, European Society of Cardiology Congress and EuroPrevent), and by forward and backward citation tracking of all eligible studies in Web of Science. No restrictions were placed on language or date of publication.

The titles and abstracts of all articles retrieved from electronic searching and other sources were screened (by BA) for eligibility against the pre-specified inclusion criteria, and full-text publications were obtained for any potentially relevant studies. Any uncertainties regarding study eligibility were resolved through discussion with two other reviewers (PG, TH) until a consensus was reached.

### Data Extraction

A standardised data extraction form (available on request) was used to extract the following: participant characteristics (gender, age, diagnosis), intervention(s) characteristics, follow-up duration, all relevant clinical outcomes reported, the type of care provided to the usual care comparison group, cardiovascular medication usage and details about the methodological quality of the trial. If multiple time points were reported, data from all which were greater than 3 months were extracted. Where multiple intervention arms were compared with usual care within a single study, each exercise arm was considered a separate intervention and data extracted accordingly.

In order to extract the individual exercise characteristics (used as co-variates in meta-regression analysis) from each included intervention in a standardised manner, a modified version of the Template for Intervention Description and Replication checklist was used [19] (Additional file 1: Table S2). Where the individual components of exercise interventions were missing or described in insufficient detail, attempts were made to locate this information in additional publications or by contacting each corresponding author (*n* = 62) via email. This process is described in further detail elsewhere [11]. While 28 authors responded to these requests and provided missing intervention details, 14 did not respond to numerous reminders. We were unable to locate contact details for a further 20 authors. A number of authors were also contacted to clarify outcome data.

### Outcomes

Our primary outcome was cardiovascular mortality. Secondary outcomes were total mortality, myocardial infarction, CABG and PCI procedures. All outcomes were assessed for the time period beginning with randomisation until the end of last reported follow-up, thereby including events which occurred before, during and after the exercise intervention period for all trials. We chose not to use a combined endpoint for analysis, as although it may have increased the statistical power required to detect treatment effects, it can also be problematic in interpretation and generate misleading conclusions, particularly about the strength of reductions in mortality [20, 21]. For this reason, the results of each outcome are presented separately.

### Risk of Bias of Included Trials

The quality of each included trial was assessed according to the Cochrane Risk of Bias Tool [22]. Trials were rated as having a low, high or unclear risk of bias for the following criteria: sequence generation (adequate randomisation methods described), concealment of allocation, study blinding (of participants, personnel and outcomes), incomplete outcome data (participant attrition) and selective outcome reporting. Blinding of participants to exercise interventions is virtually impossible; however, the clinical outcomes in our analyses are unlikely to be influenced by knowledge of group allocation. We therefore considered the criterion of blinding in terms of outcome assessment. The degree of participant attrition was assessed at the outcome, rather than the study level. For example, a substantial number of participants may not have completed the trial (high-risk attrition at study level) but the authors were able to determine from medical records whether or not these missing participants were deceased at the trial end (low-risk attrition for mortality outcome).

### Statistical Analysis

Both meta-analysis and meta-regression techniques were carried out in order to synthesise the individual trial data for each intervention and then examine the effect of individual intervention characteristics on each outcome. All comparisons of exercise-based cardiac rehabilitation versus usual care are expressed as relative risks (RRs) with 95% confidence intervals (CI) which were calculated from reported event and population data in each trial. Effects were considered statistically significant at a *p* value of <0.05. In order to minimise the bias due to missing participant data across a number of trials, the primary meta-analysis was conducted using a complete case analysis, as proposed in the Cochrane Handbook [23] and by Akl et al. [24]. This method uses, as the denominator, only those participants with complete outcome data recorded. Meta-analyses were performed in RevMan (Version 5.3, Cochrane Collaboration), with the relative risks of the outcome in each study pooled using a Mantel-Haenszel random effects model. Both the meta-analysis and meta-regression were performed using random effects models as clinical heterogeneity was expected given the wide variety of interventions and patients represented in the included trials. We tested for this heterogeneity in each meta-analysis using the Cochrane Q statistic and also calculated the *I* ^2^ to quantify the percentage of variation in effectiveness across studies which would be considered beyond a chance finding. Potential for publication bias was examined using funnel plots.

In trials with multiple intervention arms, combining these into a single group is the recommended approach for meta-analysis to avoid unit-of-analysis errors arising from correlated intervention effects. Using this approach however does not allow an investigation into the differences between treatment arms required by this analysis. Hence, we chose to use another recognised approach, dividing proportionally the number of events and total population of the shared usual care group between intervention arm comparisons, avoiding ‘double-counting’ while still allowing exploration of heterogeneity across differing interventions [23].

We also used approaches proposed by Akl et al. [25] to test the robustness of effect estimates to missing participant data (due to withdrawal, drop-out, etc.). This was done by conducting two different sensitivity analyses using plausible assumptions to impute missing participant event data for each trial. The first assumed that the incidence of events in all missing participants was the same as that observed among those followed up in the control arm of the same trial. The second approach imputed missing participant data relative to those with complete data in the same trial arm, assuming that those missing in the intervention arm had an increased event incidence of 1.5 compared to those followed up, and the control arm the same event incidence (1.0) in those with complete and missing data. We additionally performed further sensitivity analyses excluding those trials assessed at high risk of bias for missing outcome data and those published in only abstract form or as a doctoral thesis.

#### Subgroup Analyses

We conducted subgroup analyses to investigate differences in each outcome for four broad cardiac rehabilitation program comparisons which were specified a priori, based on issues raised in previous research or due to current guideline recommendations for cardiac rehabilitation and physical activity. These comparisons were (1) trials which enrolled only patients with a diagnosis of myocardial infarction vs those with mixed aetiologies [13]; (2) programs which contained ≥150 min of prescribed weekly exercise vs those with <150 min per week [26, 27]; (3) programs with a more intensive usual care arm vs programs with a standard usual care arm [12, 28]; and (4) programs that included lipid lowering statin therapy vs those that did not [13, 29]. Information about the concomitant use of lipid lowering therapy in each trial arm was extracted directly from studies where possible. Where this was not reported, we used the recruitment dates and duration of follow-up of participants to ascertain if statin use was likely, based on increasing clinical use from 1994 [30]. All subgroup differences were tested for significance using RevMan, and an *I* ^2^ statistic was also computed in order to estimate the degree of subgroup variability due to true differences rather than chance.

#### Meta-regression

The effect of individual exercise intervention characteristics on both primary and secondary outcomes was explored by using the ‘metareg’ command in Stata (Version 14, StataCorp LP) [31] to perform univariate random effects meta-regression. The median year of trial recruitment and pre-specified exercise program characteristics of intervention duration (months), exercise intensity (%HRmax), session frequency (times per week), session time (minutes), intervention provider (physician in team vs not), mode of exercise (aerobic only vs aerobic plus resistance training), delivery of exercise (group vs individual) and level of adherence to the prescribed exercise regimen (modelled as a categorical variable) were entered as co-variates. Adherence was extracted as the mean proportion of all prescribed centre and/or home-based exercise sessions completed by participants. We used established equations and tables [32, 33] to convert exercise intensities reported in varying formats across studies (e.g. Borg scale, % heart rate reserve, % VO_2max_) into one consistent format (% maximal heart rate) for all analyses.

Co-variates were considered statistically significant predictors of outcome if the *p* value associated with the RR of the meta-regression was <0.05. Multiple testing was accounted for by using the ‘permute 5000’ option in Stata to examine any variables which reached statistical significance [31]. *R* ^2^ was calculated to explain the percentage of between study variance explained by any particular co-variate. To assess whether any characteristics which displayed statistical significance in univariate analysis were independently associated with improved outcomes, we combined any co-variates with a *p* value of ≤0.2 in a multivariable model and used a stepwise backwards elimination approach to remove those which did not contribute significantly to the model.

## Results

### Trial Selection

**Fig. 1:**
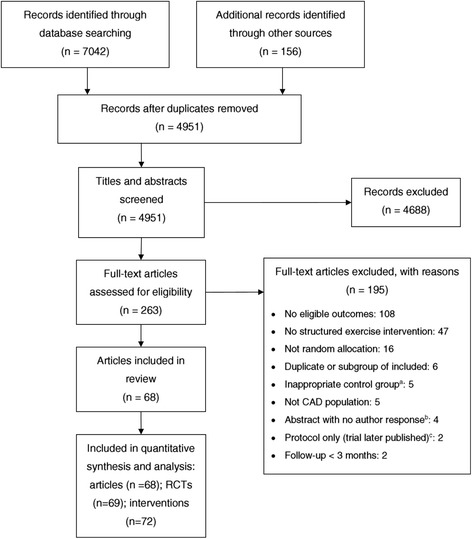
PRISMA flow diagram of the screening and selection of trials. *a* Different type of exercise or surgical intervention as comparator. *b* We were unable to assess the eligibility of one trial published as a conference abstract as the author did not respond to repeated email inquiries. Two other conference abstracts described trials with outcomes eligible for inclusion; however, these could not be included as authors were not yet ready to share their results. One further abstract was eligible for inclusion; however, the author failed to respond to requests for data (abstract references in Additional file 1: Appendix S3). *c* One of these trials was later excluded and one included. *CAD* coronary artery disease, *RCT* randomised controlled trial

Sixty-eight publications [34–101] met our inclusion criteria (see Fig. 1 for PRISMA flow chart), reporting on clinical outcomes and follow-up of 69 different trials (one publication [98] reported outcomes of a collaborative study which involved several individual trial centres). Several trials included multiple intervention arms [39, 71, 72, 87] resulting in a total of 72 individual exercise interventions.

### Trial Characteristics

Of the included 69 trials (characteristics described in Table 1), one was only reported in abstract form (for which the author provided data), one was a published doctoral thesis and the remainder were full-text publications. Most (65) were published in English, with one each in Italian and Danish and two in Russian (all of which were translated for inclusion). Publication years ranged from 1975 to 2015, and the majority of trials (59%) took place in Europe. Trial size varied from 28 to 1813 participants; however, most were small, with a median of 134 participants. The median duration of follow-up across all trials was 3 years but varied widely: the longest follow-up reported for mortality outcomes was 19 years and for myocardial infarction, CABG and PCI 10 years.

**Table 1:** Characteristics of included trials and their interventions

Trial details			Participant details				Outcomes reported	Exercise intervention	Usual care condition	Lipid-lowering therapy
Author, year [ref]	Country	Longest follow-up	*N*	Males (%)	Mean age (years)	Diagnosis		Main details (what, how, where)		
Albus, 2009 [34]	Germany	7 years	77	87	54	Angiographically documented CAD	TM, CVM, MI, CABG, PCI	Multimodal comprehensive group behavioural intervention: residential then outpatient aerobic exercise and education, 1 year	Standard cardiological care based on guidelines with frequent review	Guideline-based use of antiplatelet, statin, beta-blocker and anti-hypertensive therapy
Andersen, 1981^D^[35]	Denmark	3 years	88	100	I: 52, C: 56	Myocardial infarction	TM, CVM, MI	Outpatient aerobic (cycling, running, skipping) and resistance exercise training only, 1 year	Usual care (not specified); some trained on own	Not reported: assume no statin use due to year of study
Aronov, 2009^R^ [36]	Russia	1 year	392	94	52	Myocardial infarction, unstable angina or post-PCI	CVM, MI	Group outpatient exercise (cycling and gymnastics), 1 year	Standard cardiological care (not other specified)	Pharmacotherapy including approximately one third on statins
Belardinelli, 2001/07^a^[37, 38]	Italy	10 years	118	85	57	Post-PCI	CVM, MI, CABG, PCI	Group outpatient exercise (cycling), 6 months	Advice for daily mild physical activity but to avoid exercise training	Guideline-based pharmacotherapy but no statins started during trial
Bell, 1998^T^ [39]	UK	1 year	353	79	59	Myocardial infarction	TM	Two arms: (1) outpatient exercise and education (2) home-based walking exercise and education using the Heart Manual, 4–19 weeks	Usual care with basic advice on risk factors	Not reported: assume some statin therapy as participants recruited from 1994
Bengtsson, 1983 [40]	Sweden	1 year	171	85	56	Myocardial infarction	TM, MI	Group outpatient exercise training (aerobic high-intensity interval cycle training and callisthenics) with counselling and education, 3 months	Usual care (not specified); 4 participants undertook cardiac rehabilitation	Not reported: assume no statin use due to year of study
Bertie, 1992 [41]	UK	2 years	110	NR	53	Myocardial infarction	TM, MI, CABG#	Group outpatient aerobic exercise training (circuit-based) with reinforcement of educational information, 4 weeks	Usual care with basic advice on risk factors	Not reported: assume no statin use due to year of study
Bethell, 1990/99^UP^[42]	UK	7 years	229	100	I: 54; C: 53	Myocardial infarction	TM, CVM, MI	Group aerobic exercise training at a sports centre (circuit-based), 3 months	Usual care with exercise advice (27% exercising vigorously twice a week)	Not reported: assume no statin use due to year of study
Blumenthal, 2005 [43]	USA	4 months	134	69	63	Documented CAD and myocardial ischemia on exercise	TM, CABG, PCI#	Outpatient aerobic exercise training (cycle, jogging, stretching), 4 months	(1) Usual care, avoid formal exercise programs; (2) As (1) plus stress management training	Most patients treated with pharmacotherapy including statins
Briffa, 2005 [44]	Australia	1 year	113	73	61	Myocardial infarction, unstable angina or post revascularisation	TM, CVM, MI, CABG, PCI	Group outpatient aerobic and resistance exercise training (circuit-based) with education and counselling, 6 weeks	Standard cardiological care (9% took part in rehabilitation arm)	Individualised pharmacotherapy including statins
Byrkjeland, 2015 [45]	Norway	1 year	137	84	63	Type 2 diabetes and angiographically documented CAD	TM, MI	Group outpatient aerobic and resistance training (circuit and high-intensity interval sessions) with one home-session each week, 1 year	Standard care with general practitioner (not other specified)	Pharmacotherapy including statins for 94% of patients
Carlsson, 1998 [46]	Sweden	1 year	235	NR	62	Myocardial infarction or CABG	TM#	Group outpatient aerobic exercise (interval walking/jogging) with educational sessions (+3× pre-randomisation exercise sessions), 2–3 months	Standard cardiological care (+3 × pre-randomisation exercise sessions)	Pharmacotherapy but <30% on statins at follow-up
Carson, 1982 [47]	UK	3 years	303	100	I: 53, C: 50	Myocardial infarction	TM, MI	Exercise training at hospital gym (circuit-based), 12 weeks	Usual care (not other specified)	Not reported: assume no statin use due to year of study
DeBusk, 1994 [48]	USA	1 year	585	79	57	Myocardial infarction	TM, CVM, MI, CABG, PCI	Home-based CR via nurse-led case management which included counselling for all and aerobic exercise training for 78% of group, 1 year	Usual care with basic advice on risk factors and exercise	Physician-managed pharmacotherapy including LLT although not statin use
Dugmore, 1999 [49]	UK	5 years	124	98	52–59	Myocardial infarction	TM, MI	Aerobic (walking, jogging, cycling) and resistance exercise training, 1 year	Usual care (not other specified except to avoid formal exercise training)	Guideline pharmacotherapy but no statins used
Engblom, 1992/97 [50, 51]	Finland	5 years	228	88	54	Post-CABG	TM, CABG	Intensive exercise training (aerobic and gymnastics) and education during residential stay in outpatient centre, 3 weeks	Usual care with basic advice on risk factors and exercise	Guideline pharmacotherapy, however, is <5% using statins at any stage
Erdman, 1986 [52]	Netherlands	5 years	80	100	51	Myocardial infarction and anxiety or depression	TM, MI	Aerobic exercise (jogging), sports and relaxation training in a conventional gymnasium, 6 months	Usual care with basic advice on exercise	Guideline pharmacotherapy but no statins used
Ferreira, 2010^a^ [53]	Portugal	1 year	97	85	I: 53, C: 57	Myocardial infarction or unstable angina	TM, CVM, MI	Outpatient aerobic (cycle and treadmill) and resistance exercise training with nutritional counselling, 8 weeks	Usual care with basic advice on risk factors and exercise	Guideline-based pharmacotherapy including statins for 97%
Fletcher, 1994 [54]	USA	6 months	88	100	62	Diagnosed CHD and physical disability	TM#	Home-based aerobic exercise training on modified wheelchair treadmill with dietary instruction, 6 months	Usual care with basic advice on risk factors and exercise, same dietary instruction	Not reported: assume no statin use due to year of study
Fontes-Carvalho, 2015 [55]	Portugal	2 years	188	82	56	Myocardial infarction	TM, CVM, MI#	Outpatient aerobic (cycle and treadmill) and resistance exercise training, 8 weeks	Standard cardiological care with advice on risk factors and exercise	Guideline based pharmacotherapy including statins for 97%
Fridlund, 1992/Lidell, 1996 [56, 57]	Sweden	5 years	178	87	57	Myocardial infarction	TM, MI	‘Supportive Program’: group-based aerobic and resistance exercise, relaxation and conversation (including participant’s next-of-kin), 6 months	Usual care with basic advice on risk factors and return to work	Guideline pharmacotherapy but no statins used
Giallauria, 2008 [58]	Italy	6 months	61	72	I: 56; C: 55	Myocardial infarction	MI#	Outpatient aerobic exercise only (cycling), 6 months	Usual care with basic advice on risk factors and exercise	All medications in both groups titrated equally including statins for >70%
Haglin, 2011 [59]	Sweden	19 years	48	73	NR	Diagnosed CHD	TM	Residential group CR program for 4 weeks with aerobic exercise, counselling and education, followed by ongoing home maintenance exercise program with regular review	Usual care (not other specified)	Not reported: assume statin therapy due to follow-up years of study
Haskell, 1994 [60]	USA	4 years	300	72	57	Angiographically documented CAD	TM, CVM, MI, CABG, PCI	Individual home-based aerobic exercise (cycling and walking), diet intervention and significant medication management, 4 years	Usual care in hands of physician (not other specified)	Pharmacotherapy with LLT strategy in intervention arm although statins only available in later half of study; 31% using statins in usual care arm
Hofman-Bang, 1995/Lisspers 2005 [61, 62]	Sweden	5 years	93	84	53	Post-PCI	TM, CVM, MI, CABG, PCI	Residential group CR program for 4 weeks with aerobic exercise, counselling and education, followed by home maintenance program for 1 year with frequent nursing contacts	Usual care in hands of physician (not other specified)	Guideline pharmacotherapy including titration of statins in both arms during trial
Holmbäck, 1994 [63]	Sweden	1 year	69	97	55	Myocardial infarction	TM, MI, CABG	Outpatient group exercise training (intervals of cycling, jogging callisthenics), 12 weeks	Usual care (not other specified)	Guideline pharmacotherapy but no statins used
Kallio, 1979/Hämäläinen 1995 [64, 65]	Finland	15 years	375	80	54	Myocardial infarction	TM, CVM, MI	Outpatient group exercise (gymnastics, cycling, walking), education and counselling with ongoing home-based maintenance for 3 years: 2 sites in WHO study program	Usual care in hands of physician with basic advice on risk factors	Pharmacotherapy including non-statin LLT for 28% of intervention arm and 11% of usual care arm
Kovoor, 2006 [66]	Australia	6 months	142	87	56	Myocardial infarction with low further risk	MI, CABG, PCI	Outpatient exercise training (circuit-based), counselling and education, 5 weeks	Usual care with return to work in 2 weeks, basic advice on risk factors and exercise	Pharmacotherapy at discretion of cardiologist; < 33% on statins, similar proportions in each trial arm
Krasnitskiĭ, 2010^R^[67]	Russia	1 year	100	93	I: 55, C:54	Post-PCI	TM, CVM, PCI	Group outpatient exercise (cycling) with educational sessions, 6 weeks	Standard cardiological care (not other specified)	Maintenance pharmacotherapy for all participants; >80% on statins
La Rovere, 2002 [68]	Italy	10 years	95	100	52	Myocardial infarction	CVM, MI, CABG	Outpatient exercise training (cycling and callisthenics) and education, 4 weeks	Usual care with same education on risk factors diet and smoking as intervention group	Guideline pharmacotherapy but no statins used
Lear, 2014 [69]	Canada	16 months	78	85	I: 62, C: 58	Acute coronary syndrome, PCI or CABG with low to moderate risk	TM	Virtual, individual, home-based program with education, support, monitoring and exercise program delivered exclusively via the Internet, 4 months	Usual care with basic advice on risk factors and exercise as well as internet resources	Not reported: assume statin therapy as recruited participants from 2009 onwards
Leizorovicz, 1991 (PRECOR) [70]	France	2 years	182	100	50	Myocardial infarction	TM, CVM, MI, CABG	Outpatient group exercise training (cycling, walking, gymnastics), education and relaxation, 6 weeks	(1) Usual care of physician (not other specified); (2) as for (1) with personalised risk factor education	Guideline pharmacotherapy but no statins used
Marchionni, 2003 [71]	Italy	14 months	270	71	3 age groups: 57, 70, 80	Myocardial infarction	TM, MI	Two arms: education and counselling with either (1) outpatient exercise training or (2) home individual exercise training (both cycling, stretching, flexibility), 8 weeks	Usual care of physician with one session of risk factor education	Not reported: assume statin therapy as recruited participants from 1998 onwards
Miller, 1984 [72]	USA	6 months	203	100	52	Myocardial infarction	CVM, MI, CABG^b^	Four arms: (1) short (8 weeks), home-based exercise training (cycling); (2) long (23 weeks), home-based exercise training (cycling); (3) short, gym-based exercise training (walking); (4) long, gym-based exercise training (walking)	Usual Care (not other specified: many walking 30–45 min daily)	Guideline pharmacotherapy but no statins used
Maroto Montero, 1996/2005 [73, 74]	Spain	10 years	190	100	C: 53, I: 50	Myocardial infarction with low further risk	TM, CVM, MI, CABG, PCI	Outpatient aerobic exercise (cycling) and callisthenics training, counselling and education, 3 months	Usual care with basic risk factor advice	Pharmacotherapy at discretion of cardiologist; assume no statin therapy due to year of study
Munk, 2009 [75]	Norway	1.5 years	40	83	59	Post-PCI	MI, CABG, PCI#	Outpatient group-based high intensity interval training (cycling and running), 6 months	Usual care (not other specified)	Maintenance pharmacotherapy for all participants including statins for 95%
Mutwalli, 2012 [76]	Saudi Arabia	6 months	49	100	57	Post-CABG	TM, CVM, MI	Individual, home walking program with group outpatient education sessions and telephone support	Usual Care with basic risk factor advice	Not reported: assume statin therapy as recruited participants from 2008 onwards
Oerkild, 2012 [77]	Denmark	5.5 years	40	58	77	Older than 65 years with myocardial infarction, PCI or CABG	TM	Individual home-based walking program with support, dietary counselling and smoking education	Standard cardiological care (medication adjustment, frequent review by cardiologist)	Guideline-based pharmacotherapy including statins for all participants
Oldridge, 1991 [78]	USA	1 year	201	88	53	Myocardial infarction with depression or anxiety	TM	Outpatient group aerobic exercise training (cycle, treadmill and arm ergometry) with cognitive therapy and counselling	Usual care of physician with 50% provided referral to similar CR program	Not reported: assume no statin use due to year of study
Ornish, 1990/98 [79, 80]	USA	5 years	93	NR	I: 57, C: 62	Angiographically documented CAD	TM, CVM, MI, CABG, PCI	Individual home-based aerobic exercise training (walking), relaxation and intensive diet plan; group outpatient counselling sessions, 4 years	Usual care (not other specified)	Some participants in usual care arm began statins during trial (up to 60%). No statins started in intervention arm.
Reid, 2012 [81]	Canada	1 year	223	84	56	Post-PCI	TM, CABG#	Individual, home-based exercise training delivered via Internet with online behaviour change tutorials, 6 months	Usual care of physician with exercise advice and education booklet	Not reported: assume statin therapy as recruited participants from 2004 onwards
Román, 1983 [82]	Chile	9 years	193	90	55	Myocardial infarction	TM, CVM, MI, CABG	Outpatient group exercise training (walking, ergometry and callisthenics), 42 months	Standard cardiological care (not other specified)	Guideline pharmacotherapy but no statins used
Schuler, 1992/Niebauer, 1997 [83, 84]	Germany	6 years	113	100	54	Angiographically documented CAD	TM, CVM, MI, CABG, PCI#	Combination of individual home-based exercise (cycle) and group outpatient exercise training with diet plan and group education, 6 years	Usual care with basic risk factor and diet advice	Pharmacotherapy as required; no statins at beginning but 41% usual care and 20% intervention arm on statins at end trial
Shaw, 1981/Dorn, 1999 [85, 86]	USA	19 years	651	100	52	Myocardial infarction	TM, CVM, MI, CABG	Outpatient aerobic exercise training (circuit-based) for 8 weeks and then ongoing maintenance exercise in a gymnasium	Usual care (not specified but avoid formal exercise program)	Guideline pharmacotherapy but no statins used
Sivarajan, 1982 [87]	USA	6 months	258	80	56	Myocardial infarction	TM, CVM, CABG	Two arms: (1) individual home-based callisthenics and walking program with outpatient group education; (2) Individual home-based callisthenics and walking program only, both 12 weeks	Standard cardiological care: many exercising on own when surveyed	Not reported: assume no statin use as recruited participants before 1980
Specchia, 1996 [88]	Italy	3 years	256	91	53	Myocardial infarction	CVM, CABG, PCI	Residential-based group exercise training (cycling and callisthenics) with education for 4 weeks then home maintenance walking program	Usual care with group education sessions	Guideline pharmacotherapy but no statins used
Ståhle, 1999/Hage, 2003 [89, 90]	Sweden	4.4 years	109	80	71	Myocardial infarction or unstable angina and >65 years old	ACM, CABG, PCI#	Group-based outpatient aerobic interval training of large muscle groups to music with relaxation and option to go to education sessions, 12 weeks	Usual care with basic exercise advice and option to go to same education sessions	Increase in statin use from 10 to 20% of participants in trial after 1 year
Stern, 1983 [91]	USA	1 year	106	86	54	Myocardial infarction with decreased fitness or anxiety or depression	TM, MI, CABG	Outpatient aerobic exercise training (circuit-based high-intensity intervals), 12 weeks	(1) Group counselling and education; (2) usual care of physician but avoid formal exercise or counselling	Not reported: assume no statin use due to year of study
Toobert, 2000 [92]	USA	2 years	28	0	64	Diagnosed CAD, myocardial infarction, CABG or PCI	TM, CVM, MI#	Group aerobic exercise training, diet, relaxation and counselling at initial 7-day retreat, with decrease in continuing outpatient attendance frequency over a 2-year period	Usual care (not other specified)	Pharmacotherapy as required: 45% of usual care arm and 29% of intervention group on statin therapy
Vecchio, 1981^I^ [93]	Italy	1 year	50	100	51	Myocardial infarction	CVM, CABG	Residential exercise training (cycling and callisthenics), counselling and education, 6 weeks	Usual care and exercise less than 3 METS at home	Not reported: assume no statin use due to year of study
Vermeulen, 1983 [94]	Netherlands	5 years	98	100	49	Myocardial infarction	TM, CVM, MI	Outpatient group-based exercise training (cycling), counselling and psychological advice	Usual care (not other specified)	Not reported: assume no statin use due to year of study
Vestfold Heart Care Group, 2003 [95]	Norway	2 years	199	82	I: 54, C: 55	Myocardial infarction, unstable angina, PCI or CABG	TM, MI^UP^, CABG^UP^, PCI^UP^	Outpatient aerobic interval training of large muscle groups set to beat of music, counselling, education and relaxation (6 weeks) then 9 weeks exercise in gym	Standard cardiological care with basic risk factor advice	Guideline based pharmacotherapy for all participants including statins for >90%
Wang, 2012 [96]	China	6 months	160	83	I: 57, C: 58	Myocardial infarction	TM	Individual, home-based exercise training, education and relaxation plan for 6 weeks based on, and culturally adapted from, the ‘Heart Manual’	Usual care with basic risk factor advice	Pharmacotherapy as required including statin therapy for two thirds of participants
West, 2013 [97]	UK	9 years	1813	74	I: 64, C: 65	Myocardial infarction	TM, MI, CABG, PCI	Outpatient cardiac rehabilitation as delivered in several UK centres (all include exercise training, education and counselling), 6–8 weeks	Usual care of health system (GP review, basic risk factor education)	Pharmacotherapy including statin therapy for 60%
WHO Balatonfured, 1983 [98]	Hungary	3 years	160	100	53	Myocardial infarction	TM, CVM, MI	Group outpatient CR at local centre: exercise for most participants with educational sessions and counselling, 6 weeks	Usual care of local health system (not other specified)	Not reported: assume no statin use as recruited participants before 1980
WHO Brussels, 1983 [98]	Belgium	3 years	166	100	53	Myocardial infarction	TM, CVM, MI	Group outpatient CR at local centre: exercise for most participants with educational sessions and counselling, 8 weeks	Usual care of local health system (not other specified)	Not reported: assume no statin use as recruited participants before 1980
WHO Bucharest, 1983 [98]	Romania	3 years	129	100	53	Myocardial infarction	TM, CVM, MI	Group outpatient CR at local centre: exercise (cycling) with educational sessions and counselling, 12 weeks	Usual care of local health system (not other specified)	Not reported: assume no statin use as recruited participants before 1980
WHO Budapest, 1983 [98]	Hungary	3 years	200	100	53	Myocardial infarction	TM, CVM, MI	Group outpatient CR at local centre: exercise for most participants with educational sessions and counselling, 8 weeks	Usual care of local health system (not other specified)	Not reported: assume no statin use as recruited participants before 1980
WHO Dessau, 1983 [98]	Germany	3 years	54	100	53	Myocardial infarction	TM, CVM, MI	Group outpatient CR at local centre: exercise (cycling) with educational sessions and counselling, 6 weeks	Usual care of local health system (not other specified)	Not reported: assume no statin use as recruited participants before 1980
WHO Erfurt, 1983 [98]	Germany	3 years	119	100	53	Myocardial infarction	TM, CVM, MI	Group outpatient CR at local centre: exercise (cycling) with educational sessions and counselling, 5 weeks	Usual care of local health system (not other specified)	Not reported: assume no statin use as recruited participants before 1980
WHO Ghent, 1983 [98]	Belgium	3 years	168	100	53	Myocardial infarction	TM, CVM, MI	Group outpatient CR at local centre: exercise (cycling and gymnastics) with educational sessions and counselling, 6 weeks	Usual care of local health system (not other specified)	Not reported: assume no statin use as recruited participants before 1980
WHO Kaunas, 1983 [98]	Lithuania	3 years	115	100	53	Myocardial infarction	TM, CVM, MI	Group outpatient CR at local centre: exercise for most participants with educational sessions and counselling, 8–16 weeks	Usual care of local health system (not other specified)	Not reported: assume no statin use as recruited participants before 1980
WHO Prauge, 1983 [98]	Czech Republic	3 years	112	100	53	Myocardial infarction	TM, CVM, MI	Group outpatient CR at local centre: exercise for most participants with educational sessions and counselling, 3 years	Usual care of local health system (not other specified)	Not reported: assume no statin use as recruited participants before 1980
WHO Rome, 1983 [98]	Italy	3 years	63	100	53	Myocardial infarction	TM, CVM, MI	Group outpatient CR at local centre: exercise for most participants with educational sessions and counselling, 8 weeks	Usual care of local health system (not other specified)	Not reported: assume no statin use as recruited participants before 1980
WHO Tel Aviv, 1983 [98]	Israel	3 years	114	100	53	Myocardial infarction	TM, CVM, MI	Group outpatient CR at local centre: exercise for most participants with educational sessions and counselling, 20 weeks	Usual care of local health system (not other specified)	Not reported: assume no statin use as recruited participants before 1980
WHO Warsaw, 1983 [98]	Poland	3 years	79	100	53	Myocardial infarction	TM, CVM, MI	Group outpatient CR at local centre: exercise for most participants with educational sessions and counselling, 3 years	Usual care of local health system (not other specified)	Not reported: assume no statin use as recruited participants before 1980
Wilhelmsen, 1975 [99]	Sweden	4 years	315	89	51	Myocardial infarction	TM, CVM, MI	Outpatient exercise training (individualised high-intensity intervals, callisthenics, running, cycling), 1 year	Standard cardiological care with basic advice on physical activity	Guideline pharmacotherapy but no statins used
Yu, 2004 [100]	Hong Kong	2 years	269	76	64	Myocardial infarction or post-PCI	TM	Outpatient aerobic and resistance exercise training, vocational training and education with maintenance program offered, 8 weeks	Standard cardiological care with one education session about risk factors and physical activity	Guideline-based pharmacotherapy including statin therapy for >56% participants
Zwistler, 2008 [101]	Denmark	3 years	446	63	66	Myocardial infarction, angina, PCI or CABG	TM, MI, CABG, PCI	Outpatient group-based aerobic and resistance exercise training, education and psychological advice	Standard cardiological care (not other specified)	Guideline-based pharmacotherapy; including statin therapy for 50–60% participants

*C* control group, *CABG* coronary artery bypass graft, *CAD* coronary artery disease, *CR* cardiac rehabilitation, *CVM* cardiovascular mortality, *D* article in Danish, *GP* general practitioner, *I* intervention group, *LLT* lipid-lowering medication therapy, *MI* myocardial infarction, *NR* not reported, *PCI* percutaneous coronary intervention, *R* article in Russian, *Ref* reference, *T* article is a PhD thesis, *TM* total mortality, *UK* United Kingdom, *UP* some unpublished data obtained directly from author, *USA* United States of America, *WHO* World Health Organisation, # reported in text or flow diagram but not a primary, secondary or monitored adverse outcome of study

^a^Abstract form only

^b^Analysed as two arms (1 + 2 vs 3 + 4) due to low number of events and participants in each group

### Participant Characteristics

Of the 13,423 participants with coronary heart disease included in all trials, the mean age in individual trials ranged from 49 to 80 years, with an average age of 54 years (Table 1). The majority of participants were male (83%), with only 10 trials containing more than 25% of female participants and 26 trials (38%) comprising male participants only. A large proportion of trials (43/69; 62%) included only patients after myocardial infarction; however, those published from 1990 onward often included patients diagnosed with coronary artery disease (*n* = 8; 12%), post-PCI (*n* = 5; 7%), post-CABG (*n* = 2; 3%) or a combination of these cardiac aetiologies (*n* = 11; 16%).

### Intervention Characteristics

Despite our repeated attempts to obtain missing information [11], many details about the individual components of exercise interventions remained unknown, with 22% of interventions with missing details for at least one characteristic of session time, session frequency or exercise intensity. Hence, while these trials and interventions are included in the overall meta-analysis, they could not be entered into the meta-regression where these co-variates were missing.

#### What, How, Where and Who Details of the Interventions

An overview of the main details of each intervention is provided in Table 1. Exercise training was conducted in supervised outpatient, residential or community-based settings in the majority of interventions, with only 18% (*n* = 13) containing sessions which were conducted entirely in an unsupervised, home-based environment. Consequently, most interventions also used some form of group exercise training (80%; *n* = 53; six missing details). All 72 interventions used aerobic exercise training, with 54% (*n* = 31; 15 missing) also containing resistance training or callisthenic body-weight exercises. Interval training was used in eight interventions and circuit-based exercises in seven, while six interventions consisted only of home-based walking programs. In 53 (74%) interventions, exercise was combined with other secondary prevention strategies such as risk factor education, counselling and stress management, while the remainder provided exercise training as a standalone intervention. Allied health staff, such as physiotherapists and exercise specialists, were directly involved in the prescription and supervision of exercise training in more than half of all interventions (*n* = 32; 59%; 18 missing details), with a physician routinely contributing to exercise supervision in 16 interventions.

#### Prescribed Exercise Intervention Dose, Intensity and Adherence

Across all interventions (missing for 21 interventions), participants were reported to begin exercise training a mean of 4.8 weeks (SD 2.8) after the initial diagnosis or cardiac incident. The prescribed ‘dose’ of exercise training in these interventions varied widely (Table 2). The median duration of exercise interventions was 3 months; however, the shortest intervention lasted 3 weeks and one continued for 6 years. Exercise sessions occurred at a median frequency of three times per week, lasting a mean of 49 min (SD = 19) including warm-up and cooldown. The combination of prescribed session duration and frequency in individual interventions exceeded the guideline recommendation of 150 min of exercise per week in 67% of interventions (*n* = 38; 16 missing), with the structure of 18 interventions providing <150 min of exercise per week.

**Table 2:** Characteristics and components of exercise training interventions delivered in included trials

Author, year	Start weeks	Overall duration (months)	Session frequency (per week)	Session time (minutes)	Lowest intensity prescribed	Highest intensity prescribed	Adherence to intervention	Exercise provider
Albus, 2009	–	2.75	1	60	–	80%HR_max_ ETT	High	AH and physician
Andersen, 1981	4	12	1–2	60	–	–	–	–
Aronov, 2009	3	1.5	3	60	50%HR_max_ ETT	60%HR_max_ ETT	High	AH and physician
Belardinelli, 2001	4	6	3	53	60%VO_2max_ ETT	60%VO_2max_ ETT	High	Physician
Bell, 1998: Home	2	4.75	7	–	–	–	–	Nurse
Bell, 1998: Outpatient	6	1 or 3	1–2	20	3 mod Borg	4 mod Borg	–	AH and nurse
Bengtsson, 1983	7	3	2	30	90%HR_max_ ETT^a^	90%HR_max_ ETT^a^	High	Allied health
Bertie, 1992	3	1	2	–	–^f^	–^f^	–	Allied health
Bethell, 1990	4	3	3	30	70%HR_max predicted_^f^	85%HR_max predicted_^f^	Moderate	AH and physician
Blumenthal, 2005	–	4	3	55	70%HRR ETT	85%HRR ETT	High	Allied health
Briffa, 2005	2	1.5	3	90	60%HR_max predicted_	–	–	–
Byrkjeland, 2015	–	12	2	60	12 Borg	15 Borg^b^	Moderate	Allied health
Carlsson, 1998	4	3	2–3	60	75%HR_max_ ETT^a^	–	High	AH and nurse
Carson, 1982	6	3	2	45	–^f^	–^f^	High	AH and physician
DeBusk, 1994	–	12	5	30	60%HR_max_ ETT	85%HR_max_ ETT	–	Nurse
Dugmore, 1999	–	12	3	–	50%VO_2max_ ETT	80%VO_2max_ ETT	–	–
Engblom, 1992	7	0.75	7	60	70%HR_max_ ETT	70%HR_max_ ETT	High	Allied health
Erdman, 1986	–	6	2	75	70%HR_max_ ETT	80%HR_max_ ETT	Moderate	AH, nurse, physician
Ferreira, 2010	–	2	3	60	70%HR_max_ ETT	85%HR_max_ ETT	–	Physician
Fletcher, 1994	–	6	5	20	Not set	Not set	–	Nurse
Fontes–Carvalho, 2015	4	2	3	70	70%HR_max_ ETT	85%HR_max_ ETT	–	Allied health
Fridlund, 1992	5	6	1	30	–	–	–	Allied health
Giallauria, 2008	1	6	3	40	60%VO_2peak_ ETT	70%VO_2peak_ ETT	High	AH, nurse, physician
Haglin, 2011	–	1	5	120	–	–	–	Physician
Haskell, 1994	3	48	5	30	70%HR_max_ ETT	85%HR_max_ ETT	High	Nurse
Hofman-Bang, 1995	–	1	–	–	–	–	High	Nurse
Holmbäck, 1994	8	3	2	35	70%HR_max_ ETT^a^	85%HR_max_ ETT^a^	High	Allied health
Kallio, 1979	2	3	3	60	13 Borg	13 Borg	High	AH and physician
Kovoor, 2006	6	1.25	2–4	60	70%HR_max_ ETT^f^	85%HR_max_ ETT^f^	–	Nurse
Krasnitskiĭ, 2010	–	1.5	3	60	50%HR_max_ ETT	60%HR_max_ ETT	High	Physician
La Rovere, 2002	4.5	1	5	30	75%HR_max_ ETT	95%HR_max_ ETT^d^	High	–
Lear, 2014	–	4	3–5	Customised	–	–	–	Nurse
Leizorovicz, 1991 (PRECOR)	4	1.5	3	25	80%HR_max_ ETT	80%HR_max_ ETT	High	–
Marchionni, 2003	4	2	5	30	70%HR_max_ ETT	85%HR_max_ ETT	High	Allied health
Miller, 1984: Gym	3	2 or 5.75	3	60	70%HR_max_ ETT	85%HR_max_ ETT	Moderate	Nurse and physician
Miller, 1984: Home	3	2 or 5.75	5	30	70%HR_max_ ETT	85%HR_max_ ETT	High	Nurse
Maroto Montero, 1996/2005	2	3	3	60	75%HR_max_ ETT	85%HR_max_ ETT^d^	–	–
Munk, 2009	2	6	3	60	60%HR_max_ ETT^c^	90%HR_max_ ETT^c^	–	Allied health
Mutwalli, 2012	1	6	7	30	–	–	–	–
Oerkild, 2012	–	1.5	6	30	11 Borg	13 Borg	–	Allied health
Oldridge, 1991	6	2	2	50	65%HR_max_ ETT	65%HR_max_ ETT	High	AH and physician
Ornish, 1990	–	48	6	30	50%HR_max predicted_	80%HR_max predicted_	–	–
Reid, 2012	1	6	–	–	–	–	–	Allied health
Román, 1983	8	42	3	30	70%HR_max_ ETT	70%HR_max_ ETT	High	Allied health
Schuler, 1992	–	72	2	60	75%HR_max_ ETT	75%HR_max_ ETT	Moderate	–
Shaw, 1981	–	2	3	60	85%HR_max_ ETT^g^	85%HR_max_ ETT^g^	High	–
Sivarajan, 1982: both arms	1	3	7	60	MET table	MET table	–	AH and nurse
Specchia, 1996	5	1	5	30	75%HR_max_ ETT	75%HR_max_ ETT	High	–
Ståhle, 1999	3	3	3	50	50%HR_max_ ETT^d^	80%HR_max_ ETT^d^	High	Allied health
Stern, 1983	–	3	3	60	85%HR_max_ ETT^~a^	85%HR_max_ ETT^~a^	High	AH and physician
Toobert, 2000	–	15	2	60	11 Borg	13 Borg	High	Allied health
Vecchio, 1981	4	1.5	6	45	75%HR_max_ ETT	75%HR_max_ ETT	–	Physician
Vermeulen, 1983	4	2	5	–	–	–	–	Physician
Vestfold Heart Care Group, 2003	1–4	3.75	2	45	11 Borg^e^	15 Borg^e^	High	Allied health
Wang, 2012	1	1.5	–	–	–	–	–	Nurse
West, 2013	–	1.75	1–2	60	–	–	Moderate	Allied health
WHO Balatonfured, 1983	5–6	1.5	–	–	–	–	High	–
WHO Brussels, 1983	8	2	–	–	–	–	Moderate	–
WHO Bucharest, 1983	12	3	–	30	60%PWC_max_ ETT	70%PWC_max_ ETT	Moderate	–
WHO Budapest, 1983	4–6	2	–	–	–	–	Moderate	–
WHO Dessau, 1983	6	1.5	7	30	60%HR_max_ ETT	70%HR_max_ ETT	High	Allied health
WHO Erfurt, 1983	5	1.25	7	30	60%HR_max_ ETT	70%HR_max_ ETT	Moderate	Allied health
WHO Ghent, 1983	6–8	1.5	3	60	70%HR_max_ ETT	80%HR_max_ ETT	Moderate	Allied health
WHO Kaunas, 1983	6	2–4	–	–	–	–	High	–
WHO Prauge, 1983	12	36	–	–	–	–	High	–
WHO Rome, 1983	8	2	–	–	–	–	Moderate	–
WHO Tel Aviv, 1983	10–12	5	–	–	–	–	High	–
WHO Warsaw, 1983	4–6	36	–	–	–	–	High	–
Wilhelmsen, 1975	12	12	3	30	80%HRR ETT^a^	80%HRR ETT^a^	Moderate	Allied health
Yu, 2004	6	2	2	60	65%HRR_predicted_	85%HRR_predicted_	–	Allied health
Zwistler, 2008	–	1.5	2	90	60%HRR ETT	85%HRR ETT	–	Allied health

*AH* allied health practitioners including physiotherapists, exercise specialists and occupational therapists, *Borg* Borg rating of perceived exertion 6–20 scale, *d* progressed to this higher intensity by the end of the intervention period, *ETT* achieved during graded exercise tolerance test or functional capacity test, *HR*_*max*_ maximal heart rate, *HRR* heart rate reserve, *MET* metabolic equivalent, *mod Borg* modified 0–10 Borg scale, *predicted* value predicted from age adjusted equation, *PWC* peak work capacity, *VO*_*2max*_ maximum oxygen consumption, *VO*_*2peak*_ peak oxygen consumption

^a^Performed as interval training (no further information provided)

^b^Performed as interval training (20–60 s on and 20 s off for 3–4 min sets)

^c^Performed as interval training (3 mins at 80–90% HR_max_ETT, 4 min at 60–70% HR_max_ETT)

^d^Performed as interval training (3–4 min at highest intensity, 3–4 min at lowest intensity)

^e^Performed as interval training (3–4 min at 13 Borg scale progressing to 15 Borg scale by end of intervention period, 3–4 min at 11 Borg scale)

^f^Performed as circuit training (no further information provided)

^g^Performed as circuit training (4 min on each modality followed by 2 min rest)

Where reported (*n* = 51, Table 2), exercise intensity was most often prescribed using a target range, rather than a single value for the level of effort required. Given the range of prescribed intensities within a single intervention, some degree of individualisation is assumed, along with differing rates of progression to the top of the prescribed range. This range was most commonly based on a percentage of the actual peak heart rate achieved during symptom-limited maximal exercise testing; however, in four cases, it was based on an age-predicted maximal heart rate. We also noted exercise intensity prescribed in several other formats including the Borg scale (*n* = 6), METs (*n* = 1), or peak VO_2_ (*n* = 3) or work capacity (*n* = 1) measured during symptom-limited exercise testing. Once converted to the same scale, the mean minimum exercise intensity prescribed across all interventions was 68% of the maximal heart rate, increasing up to a mean of 80% at the top of the prescribed range. Overall, the majority of interventions prescribed levels of exercise which could be classified as either moderate-to-vigorous (*n* = 6; 12%), vigorous (*n* = 28; 55%) or vigorous-to-high (*n* = 5; 10%) in intensity [33]. Interventions prescribing interval training generally took the format of vigorous (rather than high or maximal) intervals lasting 3–4 min interspersed with 2–3 min of active or passive recovery (refer to Table 2 footnotes for details).

Adherence to the prescribed exercise regimen was reported for 46 interventions, and ranged from 60–100%. For supervised interventions (*n* = 42), this information was routinely collected by program or trial staff based on attendance, while unsupervised interventions (*n* = 4) relied on self-reported participant exercise logs, or data collected via the phone each week. Almost three quarters of interventions (*n* = 33) reported levels of adherence which we classified as high (≥75% all prescribed exercise sessions completed), with the remainder displaying moderate adherence levels (50–74% of sessions completed).

### Usual Care Comparisons

The care provided in the usual care arm of trials varied greatly (Table 1). For many comparisons (*n* = 31; 41%), the exact nature of care received in this arm was unspecified, described only as usual care in the hands of a physician or local health service. Participants in the control arm of 10 other comparisons were reported to receive ‘standard cardiological care’, which usually consisted of guideline-based treatment, including regular cardiology or nursing review, and medication titration. The remaining 31 control comparison groups also received either usual or cardiological care, with the addition of risk factor and/or general physical activity advice (e.g. eat less fat, walk daily). Unlike home-based intervention arms, these control arm interventions did not take the form of a prescribed and structured program but consisted of printed or on-line material, a single education session, face-to-face discussion or personalised advice. The first two types of control arms were classified as ‘usual care’ in this review, whereas we considered participants in the latter type of trials to have been delivered ‘usual care plus lifestyle advice’ in the control arm.

Guideline-based medication regimens which included statin therapy were reportedly used at baseline, during the trial, or throughout follow-up, in 24 (34%) of the control arm comparisons. In a further seven comparisons (10%), statin therapy was not explicitly reported but probable given the dates of participant recruitment. Lipid-lowering therapy was either not begun in the control arm, or reported as not used, in 17 comparisons (24%) and was unlikely used in a further 24 comparisons with recruitment and follow-up periods preceding 1994. Overall, this resulted in statin use in the control arm (matched with comparable use in the intervention arm) for 40% of comparisons, 56% of comparisons without statin use in either arm and three trials [46, 48, 60] in which the intervention arm included a specific pharmacological lipid-lowering strategy compared to the control arm (Table 1).

### Publication Bias and Quality Assessment

#### Publication Bias

The funnel plot for the primary outcome of cardiovascular mortality did not suggest asymmetry or publication bias (Additional file 1: Figure S4a). Similarly, evidence of publication bias was not observed for the outcomes of total mortality, myocardial infarction or CABG. The funnel plot for the PCI outcome however displayed possible asymmetry, suggesting the absence of small studies with favourable effects from the analysis (Additional file 1: Figure S4b).

#### Allocation: Random Sequence Generation and Concealment

The reported methods used for random sequence generation were considered to be at low risk of selection bias in 34 trials (49%). One study [59] was rated to be at a high risk of bias, as while initial randomisation occurred from all referrals using a number table; when a participant allocated to the intervention arm was not able to take part in the treatment clinic, a new patient was randomly selected from the referral population to be a case. In the remaining 34 interventions, while randomisation was reported, the risk attributable to sequence generation was unclear due to a lack of information. The allocation sequence was reported as adequately concealed in only a quarter of trials (*n* = 17). The remaining 52 trials did not provide enough information to make a judgment about the risk of bias in this domain.

#### Blinding: Participants, Personnel, and Outcome Assessment

Due to the inherent nature of exercise training, blinding of participants and providers to the intervention received did not occur in any trial. However, in seven trials (10%), the outcome assessors were blinded to participant group, and a further 23 (33%) used methods of outcome assessment (e.g. medical records, registry data) which would be unlikely to suffer from bias due to lack of blinding and we judged these trials to be at low risk of bias. Whether or not blinding of outcome assessment occurred or objective data sources were used was unclear in 36 trials (52%). Three trials which explicitly stated that the study and outcome assessment were unblinded were rated at high risk of bias.

#### Incomplete Outcome Data

In terms of mortality outcomes, 11 trials (16%) did not account for a loss of participant outcome data exceeding 15% and were consequently rated as at a high risk of attrition bias. These trials had a mean of 25% missing data, with one [79] unable to account for outcomes of almost 50% of randomised participants, who later declined to consent to study procedures (although this dropout was equal across both trial arms). In regards to the other outcomes of myocardial infarction, CABG and PCI, 19% of trials (*n* = 13) were rated at high risk of attrition bias.

Overall, poor reporting meant that 86% of trials were rated as an unclear risk of bias in one or more of the above domains (Additional file 1: Table S5). Eight trials (12%) were assessed to be at low risk of bias across all four domains, while 17 (25%) were rated at high risk of bias for one or more domains, most usually incomplete outcome data. Additionally, bias due to selective outcome reporting could only be assessed for five of the included studies [55, 71, 75, 81, 101] in which all pre-specified primary and secondary outcomes in the protocols were reported in the published trials. Consequently, most information in this review is from studies rated at unclear risk of bias, and the proportion of studies at high risk of bias for missing outcome data needs to be considered when interpreting the results (Additional file 1: Figures S5a and S5b).

### Primary Outcome: Cardiovascular Mortality

**Fig. 2:**
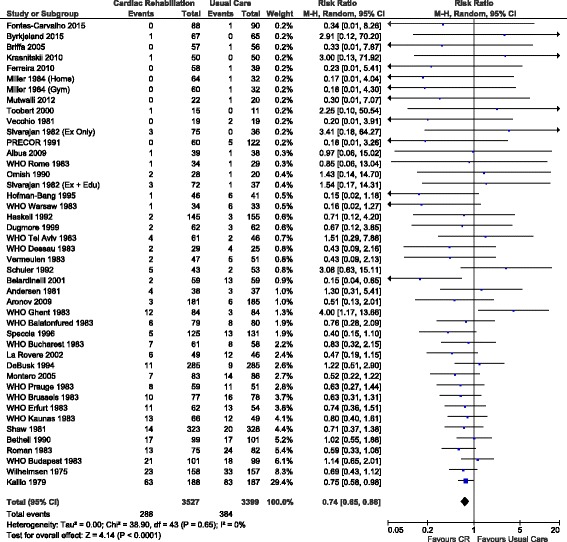
Forest plot of the effect of exercise-based cardiac rehabilitation (vs usual care) on cardiovascular mortality across all types of interventions, *CI* confidence interval, *CR* cardiac rehabilitation

Cardiovascular mortality, reported in 31 trials (comprising 44 different interventions and 6926 participants), showed a statistically significant reduction with exercise-based cardiac rehabilitation compared to usual care over a follow-up period of 10 years in length (RR 0.74, 95% CI 0.65 to 0.86, *p* < 0.0001) (Fig. 2). When considering follow-up out to 19 years (additional information provided in two trials), a smaller reduction in risk was observed (RR 0.82, 95% CI 0.73 to 0.91, *p* = 0.0004). No evidence of heterogeneity was observed in either analysis (*I* ^2^ = 0%). Sensitivity analysis also demonstrated the effect estimates to be robust to missing participant data (Additional file 1: Table S6a) and publication type (Additional file 1: Table S7a).

#### Analysis of Intervention Components

Subgroup analyses with interventions stratified based on population diagnosis (*I* ^2^ = 0%, *p* = 0.60 for interaction effect), minutes of prescribed exercise per week (*I* ^2^ = 0%, *p* = 0.81), type of usual care (*I* ^2^ = 0%, *p* = 0.72) or the use of lipid-lowering therapy (*I* ^2^ = 0%, *p* = 0.60) did not demonstrate any differences in effect on cardiovascular mortality (Additional file 1: Table S8a).

**Fig. 3:**
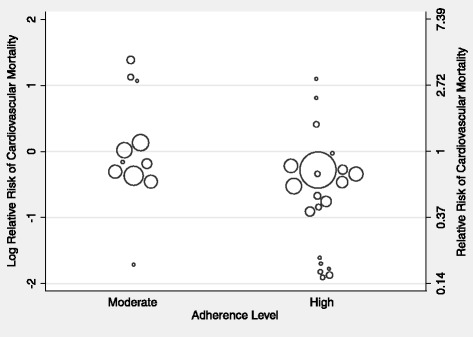
Relationship between the reported level of exercise intervention adherence and the relative risk of cardiovascular mortality compared to usual care. Each intervention is represented by a *circle*; the size of the *circle* is proportional to the number of participants undertaking that intervention. A log RR of >0 represents an increase in risk and <0 a decrease

Univariate meta-regression analysis found no significant effect of the year of recruitment to the trial, exercise mode and exercise provider; how the exercise was delivered; or the prescribed intervention duration, session frequency, time or intensity on cardiovascular mortality outcomes (Additional file 1: Table S9a). A significant relationship was however observed between the level of adherence to the exercise intervention and cardiovascular mortality (Fig. 3), with a 28% reduction in relative risk (RR 0.72, 95% CI 0.52–0.99, *p* = 0.045) observed for those comparisons reporting high levels of adherence to the prescribed exercise intervention compared to those reporting only moderate levels of adherence. No other co-variates met the pre-specified *p* value cut-off for entry into the multivariate model.

### Secondary Outcome: Total Mortality

**Fig. 4:**
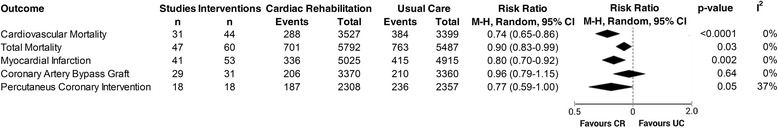
The effect of exercise-based cardiac rehabilitation versus usual care on coronary heart disease outcomes. Diamonds represent the pooled summary estimate of random effects Mantel-Haenszel meta-analysis for each outcome. *CR* cardiac rehabilitation, *UC* usual care

The pooled meta-analysis found a 10% reduction in overall mortality risk with exercise-based cardiac rehabilitation compared to usual care over a follow-up period of 10 years (Fig. 4). When extending the follow-up period out to 19 years, no demonstrable reduction in overall mortality was observed (RR 0.97, 95% CI 0.90 to 1.04, *p* = 0.37). These intervention effects were also less robust to missing data with the sensitivity analysis suggesting a trend towards non-significance (Additional file 1: Table S6b). No evidence of heterogeneity was observed in either analysis (*I* ^2^ = 0%).

#### Analysis of Intervention Components

Subgroup analyses with interventions stratified based on population diagnosis, minutes of prescribed exercise per week and the type of usual care did not demonstrate any differences in effect on total mortality (Additional file 1: Table S8b). Moderate heterogeneity however (*I* ^2^ = 48%, *p* = 0.15 for interaction) was observed among subgroups divided by use of concomitant lipid-lowering therapy, suggesting this may account for some of the variability in the effect observed. The trend observed was for exercise-based cardiac rehabilitation interventions to have a reduced effect in the presence of lipid-lowering therapy, although this effect did not reach significance.

Univariate meta-regression analysis found that exercise adherence was the only co-variate which significantly predicted total mortality outcomes (Additional file 1: Table S9b). A 19% reduction in relative risk (RR 0.81, 95% CI 0.66–0.996, *p* = 0.042) was observed for those comparisons reporting high levels of adherence to the prescribed exercise intervention compared to those reporting only moderate levels of adherence. While several co-variates met the pre-specified *p* value cut-off for entry into the multivariable model, after backwards elimination of non-significant predictors, only adherence remained.

### Secondary Outcome: Myocardial Infarction

Random effects meta-analysis found that exercise-based cardiac rehabilitation produced a 20% reduction in myocardial infarction events compared to usual care (Fig. 4). This effect was also robust to missing data and publication type in sensitivity analysis (Additional file 1: Table S6c; Additional file 1: Table S7c), and no evidence of heterogeneity was observed (*I* ^2^ = 0%).

#### Analysis of Intervention Components

Subgroup analyses with interventions stratified based on population diagnosis, minutes of prescribed exercise per week and type of usual care did not demonstrate any differences in effect on myocardial infarction events (Additional file 1: Table S8c). Low to moderate heterogeneity (*I* ^2^ = 33%, *p* = 0.23 for interaction effect) was observed among subgroups divided by use of concomitant lipid-lowering therapy, which was largely due to the difference in effect observed in the subgroup of two trials providing statin therapy as part of the intervention arm.

**Fig. 5:**
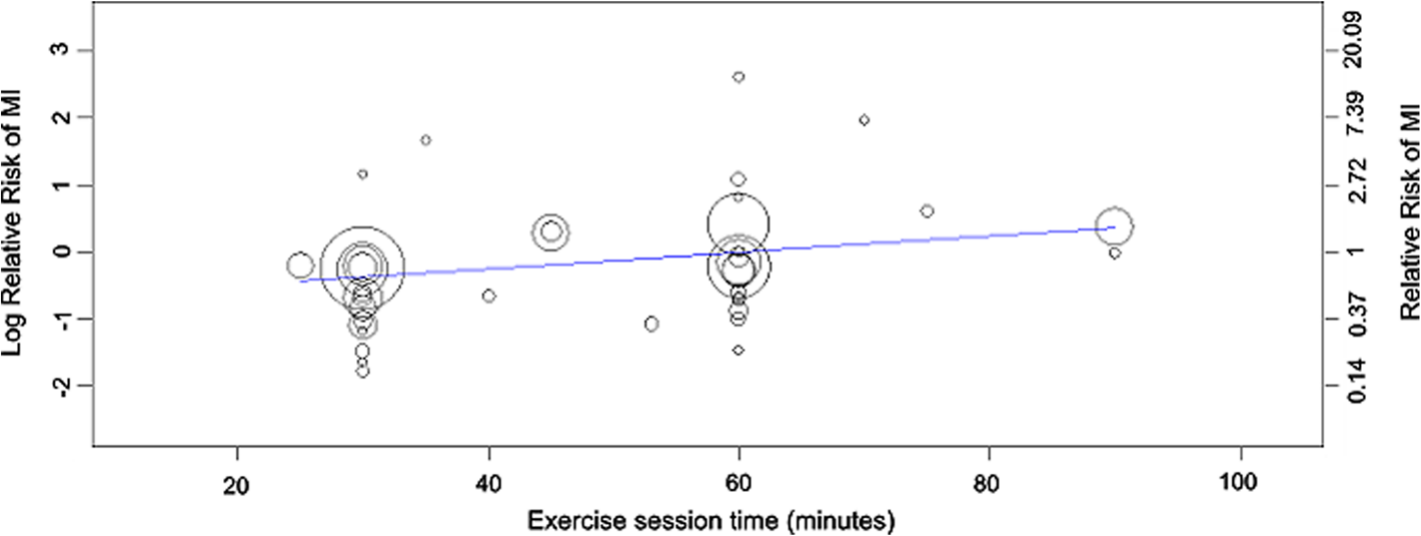
Relationship between the prescribed time for exercise training each session and the relative risk of myocardial infarction compared to usual care (*p* = 0.011). Each intervention is represented by a *circle*; the size of the *circle* is proportional to the number of participants undertaking that intervention. A log RR of >0 represents an increase in risk and <0 a decrease

Univariate meta-regression analysis found only one co-variate that significantly predicted myocardial infarction outcomes (Additional file 1: Table S9c). A significant positive relationship was observed between the total time prescribed for exercise each session and the risk of myocardial infarction (Fig. 5). That is, for every 1 min increase in time (between 25 and 90 min), the relative risk of myocardial infarction with the exercise intervention vs usual care increased by 1%. While several co-variates met the pre-specified *p* value cut-off for entry into the multivariable model, after backwards elimination of non-significant predictors, only exercise time remained. Due to a moderate pairwise correlation between session time and session frequency (*r* = −0.512), some potential multicollinearity may have been observed between these two co-variates in the multivariable analysis. However, repeating the analysis accounting for this fact did not produce different results about potential predictors of myocardial infarction outcomes.

### Secondary Outcome: Coronary Artery Bypass Grafting

Random effects meta-analysis found no demonstrable reduction in the number of participants who required coronary artery bypass grafting (CABG) in the exercise-based cardiac rehabilitation interventions compared to usual care (Fig. 4). Sensitivity analysis for missing outcome data did not change this effect estimate (Additional file 1: Table S6d), and no evidence of heterogeneity was observed (*I* ^2^ = 0%).

#### Analysis of Intervention Components

Subgroup analyses with interventions stratified based on minutes of the prescribed exercise per week, the type of usual care or the use of lipid-lowering therapy did not find any differences in effect on the number of CABG procedures performed (Additional file 1: Table S8d). However, a significant interaction was found between the population diagnosis and outcome, with exercise interventions undertaken by participants with mixed aetiologies, demonstrating a greater reduction in CABG event rate compared to usual care than those which comprised purely myocardial infarction patients (*p* = 0.04, *I* ^2^ = 76%).

Univariate meta-regression analysis did not find any co-variates to have an effect on CABG outcomes (Additional file 1: Table S9d). Prescribed intervention duration was the only co-variate which met the pre-specified *p* value cut-off for entry into the multivariate model; consequently, multivariate meta-regression was not performed for this outcome.

### Secondary Outcome: Percutaneous Coronary Intervention

The pooled meta-analysis found no difference between exercise-based cardiac rehabilitation and usual care in reducing PCI events (Fig. 4), with some possibly important heterogeneity observed (*I* ^2^ = 37%, *p* = 0.06). Sensitivity analysis for missing outcome data did not change this effect estimate or substantially reduce the degree of heterogeneity (Additional file 1: Table S6e). Excluding trial data obtained only in abstract form reduced heterogeneity without substantially changing the effect estimate (Additional file 1: Table S7d).

#### Analysis of Intervention Components

Subgroup analyses with interventions stratified based on population diagnosis, type of usual care or minutes of prescribed exercise per week did not demonstrate any differences in effect on the number of PCI procedures performed (Additional file 1: Table S8e). Some non-significant heterogeneity (*I* ^2^ = 21%, *p* = 0.28) was observed for subgroups stratified by use of lipid-lowering therapy. However, stratifying the overall meta-analysis based on this factor could not account for the overall heterogeneity observed.

**Fig. 6:**
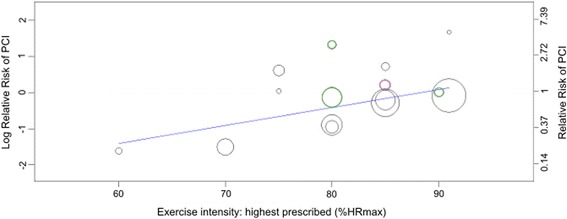
Relationship between the highest intensity of exercise prescribed (as a percentage of maximal heart rate) and the relative risk of percutaneous coronary intervention compared to usual care (*p* = 0.047). Each intervention is represented by a *circle*; the size of the *circle* is proportional to the number of participants undertaking that intervention. A log RR of >0 represents an increase in risk and <0 a decrease. *Circles* coloured *green* represent interventions for which the highest intensity was only prescribed for brief periods during interval training. *Circles* coloured *purple* represent interventions for which the highest intensity was only prescribed during the work periods of circuit training

Univariate meta-regression analysis found no significant effect of the year of recruitment to the trial, exercise mode, format of exercise training or prescribed intervention duration, session frequency or time on PCI outcomes (Additional file 1: Table S9e). A significant positive relationship was however observed between the highest level of intensity prescribed and the risk of PCI (Fig. 6). That is, for every 1% increase in maximal heart rate prescribed (between 60 and 91% HR_max_), the relative risk of PCI with the intervention compared to usual care increased by 5%. Additionally, in interventions where the team providing exercise training included medical practitioners, as opposed to nursing or allied health staff alone, the risk of PCI was significantly decreased (RR 0.30, 95% CI 0.14–0.62, *p* = 0.004). None of the co-variates which met the pre-specified *p* value cut-off for entry into the multivariate model remained after backwards elimination of non-significant predictors.

## Discussion

This meta-analysis demonstrates exercise-based cardiac rehabilitation to be effective in reducing total and cardiovascular mortality, as well as myocardial infarction, in participants with coronary heart disease. This effect was largely consistent across subgroups of patients who received various types of usual care, more or less than 150 min of exercise per week, and of differing cardiac aetiologies. Additionally, the effectiveness of cardiac rehabilitation to reduce PCI procedures displayed borderline significance, which may be important given that the funnel plot displayed a possible absence of small studies reporting positive effects.

While the largest proportion of included trials and interventions remained traditional group-based programs of supervised aerobic (and often resistance) training, our review also found an expanding range of interventions including home walking programs, high-intensity interval training, case management and Internet technologies. Consequently, the prescribed dose of exercise training varied widely, with some interventions offering a substantially greater volume of training than others. However, we were unable to demonstrate evidence for the effectiveness of any one specific exercise component, such as intensity, frequency, session time or type, on reducing mortality outcomes. A relationship was observed however between increasing levels of adherence to exercise training and a reduction in subsequent mortality. In contrast, we found a detrimental effect of increasing the prescribed exercise time and intensity on myocardial infarction and PCI outcomes respectively.

Overall, the results of our pooled analyses are generally consistent with previous meta-analyses of exercise-based cardiac rehabilitation. These studies also found a significant reduction in the relative risk of cardiovascular mortality, with a similar magnitude of effect observed in the most recent analyses [8, 15], which also included patients of mixed aetiologies. The inability of cardiac rehabilitation to significantly reduce the frequency of CABG and PCI events was also reported in these two earlier studies. The previously observed effects of cardiac rehabilitation on myocardial infarction and total mortality have however been mixed.

While earlier meta-analyses found significant reductions in total mortality of up to 20–30% [12, 15, 102, 103], the most recent review [8], which represented a broader range of participants and trials conducted in the modern treatment era, failed to find such a benefit. While we observed a significant 10% reduction in total mortality in our sample, this effect was less robust to missing data in sensitivity analysis and provides some evidence to support the hypothesis that the effect of cardiac rehabilitation on total mortality may be attenuated when considering a wider range of participants in the modern treatment era.

### Exercise-Based Cardiac Rehabilitation in the Modern Treatment Era

It has been previously suggested that the benefits of exercise-based cardiac rehabilitation over usual care may be incremental in the modern era, with the advent of lipid-lowering therapy and optimal medical treatment [8, 97]. Previous meta-analyses [8, 10, 15, 102] have examined this hypothesis with subgroup analysis comparing the outcomes of trials published before and after 1994/95, with this date acting as a surrogate for changing practices in treatment reported after this time [30]. While these studies found no significant subgroup differences and consequently claimed the benefits of cardiac rehabilitation to be maintained in the modern era, the use of publication date in this manner has several limitations. Firstly, it is a crude measure of when a trial was conducted and may not accurately reflect treatment practices at the time of publication. Trials with long recruitment and follow-up periods, along with delays in publication, mean that not all post-1994 trials were necessarily conducted in the modern treatment era. For example, one trial published in 2002 [68] reported a 10-year follow-up of patients recruited in 1984 and 1985 and was conducted wholly in the pre-statin era. Additionally, at least one study published post-1995 [37] explicitly excluded the use of lipid-lowering therapy. Including these trials in their respective year-based subgroups may not provide a true reflection of changes in care. Furthermore, even in the modern treatment era, the level of usual care provided to the control arm can vary widely, from follow-up with a physician to educational packages or sessions and targeted guideline advice about lifestyle modification. This is an important consideration as Janssen et al. [28] have previously shown that there may be a dilution of effectiveness for secondary prevention interventions when compared to control groups where usual care is optimal.

Therefore, to assess the impact of cardiac rehabilitation treatment in the modern era, we examined the effects of both usual care and lipid-lowering therapy separately, as well as performing meta-regression analysis based on the median year of reported trial recruitment. Subgroup analysis was not able to replicate the findings of Janssen, with no significant differentiation in the effects of cardiac rehabilitation with the varying levels of usual care provided. This seems to support the importance of providing structured exercise training even in the presence of other lifestyle change measures, such as physical activity advice, education or counselling. This is an important consideration given that the level of usual care provided in clinical practice may often be less ideal than that delivered in clinical trials. While subgroup analysis was not able to display a significantly different effect of cardiac rehabilitation in programs with lipid-lowering therapy compared to those without, we did observe some evidence that total mortality was less likely to be reduced by cardiac rehabilitation in the presence of lipid-lowering therapy. This was more pronounced when excluding those studies with a specific lipid-lowering strategy in the intervention arm (*p* for subgroup differences = 0.07, *I* ^2^ = 70%).

While this attenuation of effect may be due to statin therapy alone, it is more likely confounded by other improvements in medical management and patient demographics in these later trials which included lipid-lowering therapy. Essentially, as hypothesised, statins may be acting as a surrogate marker for improved medical management over time. This argument is in agreement with the most recent Cochrane review on the topic where a trend was observed via meta-regression for smaller reductions in total mortality with cardiac rehabilitation trials published in more recent years [8]. However, our analysis using a more clinically meaningful co-variate for trial year found no trend or significant association between the median year of participant recruitment to the trial and the subsequent risk of mortality. Together, these findings suggest that while pharmaceuticals may affect the ability of cardiac rehabilitation to reduce overall mortality to some extent, the importance of this intervention in reducing clinical events in the modern treatment era does not appear to be greatly diminished.

### The Impact of Exercise Intervention Characteristics and Prescribed ‘Dose’

Our findings extend those of previous meta-analyses of exercise training in cardiac rehabilitation, which have also found no effect of prescribed exercise dose (reported as program duration × session time × session frequency) on clinical outcomes [8, 10, 15]. However, analysing the effect of exercise interventions based on such a crude overall measure of ‘dose’ may have acted to mask the effects of the individual components in these earlier analyses, and none took into account varying exercise intensities. Additionally, the level of poor intervention reporting we observed in the majority of trials would have hampered the ability of researchers to obtain all three elements required for the calculation of dose (e.g. the most recent Cochrane review [8] was only able to examine the effect of 18 doses of exercise on cardiovascular mortality out of 24 trials). Our use of individual exercise components, along with efforts to obtain missing descriptions, meant that we were able to explore a greater range of dose comparisons in analysis, with increased power to detect a true effect. The finding that few differences in effect were present, despite these improvements in methodology, supports the conclusions of the original meta-analyses. Additionally, they are consistent with a systematic review which found no effect of exercise session frequency or duration on improvements in fitness in cardiac rehabilitation patients [18].

It could be argued that energy expenditure expressed as either MET-hours or kilocalories would have provided the most useful measure of exercise dose accounting for various combinations of intensity, frequency and duration of exercise sessions prescribed within an intervention. However, the combination of missing intervention characteristics and a lack of knowledge about baseline VO_2_ values in the majority of included trials meant that we were unable to obtain the necessary information to calculate energy expenditure within most individual interventions. Consequently, we could not examine the relationship between this measure of exercise dose and clinical outcomes. Improving the reporting of intervention characteristics and baseline exercise data in future trials would help to address this issue.

While meta-regression analyses provide a large amount of evidence based on indirect comparisons of exercise training interventions within the cardiac population, relatively few trials exist which provide direct, head-to-head comparisons of variations in individual exercise components. Our findings however are largely in agreement with this small body of literature which has found no effect of interventions with differing durations [104], frequencies [105, 106], session times [107] or proportions of hospital-based sessions [108–110], on fitness, risk factors or quality of life. While avoiding the potential aggregation bias of subgroup analysis and allowing a more robust comparison of intervention characteristics, these trials are unfortunately limited by short follow-up periods and generally report only on risk factors or biological parameters, making it difficult to assess their impact on the long-term clinical outcomes observed in our analysis.

In our analysis, we did not observe higher intensity interventions to be any more effective than lower intensity ones in reducing mortality or preventing myocardial infarction in the long term. This differs to other research which suggests that increased exercise intensity is an important factor in achieving superior outcomes in patients with cardiovascular disease. Systematic reviews have shown high-intensity interval training (HIIT) to be better than moderate-intensity continuous exercise training at improving fitness (VO_2_ peak) in patients with coronary heart disease [111, 112] or lifestyle-induced chronic disease [113]. Increased exercise intensity was also associated with increased gains in fitness in a recent systematic review [18] and in a large cohort of cardiac rehabilitation patients [114]. However, our review included only eight trials which specifically used interval training protocols, and the intensities prescribed during work intervals were generally lower than those observed in the HIIT literature. Additionally, all trials included in our analysis compared changes in clinical endpoints rather than fitness. While cardiorespiratory fitness is recognised as an important prognostic factor for mortality [115, 116] and is associated with decreased incidence of myocardial infarction and revascularisation in patients with CAD [117], a direct link between exercise intensity and clinical endpoints is yet to be made, as most trials are limited by short-term follow-up (4 months or less) and are underpowered to detect changes in clinical endpoints. One trial which did report an extended follow-up period of 4 years [118] found no difference in the rate of recurrent myocardial infarction (fatal and non-fatal) between patients randomised to low or high intensity exercise.

We did however observe evidence of a statistically significant relationship between the highest intensity of exercise prescribed to participants and an increased risk of PCI events. While we were unable to find similar reports of this phenomenon in previous research, one possible explanation may be that higher intensity exercise causes higher myocardial oxygen demands (as a result of increasing heart rate, myocardial contractility, ventricular work and blood pressure) which if not met, may elicit myocardial ischaemia not otherwise observable at lower intensities [119, 120]. If observed, these symptoms may trigger further examination and invasive intervention, given that trial participants are reviewed regularly, and that PCI (like CABG) is a physician-driven endpoint which may be prone to clinically subjective decisions [121] (an elective rather than a spontaneous event like death or infarction). Additionally, PCI (as opposed to CABG) would most likely be considered the primary method of revascularisation in this population, given that most of those enrolled in the trials would be at low subsequent cardiovascular risk [122]. It is also important to note that only three of the trials reporting PCI outcomes prescribed higher training intensities in an interval format [75, 89, 95], and it may be that periods of intermittent recovery (as used in HIIT) are needed to mediate the potential negative effect of higher intensity exercise.

Why an increase in the risk of MI should occur with longer prescribed session times is also not clear. One previous trial in coronary patients has found exercise durations extended to 60 min blunted the beneficial effects on antioxidants and vasculature observed with 30 min of training [123]. Conversely, 40 and 60 min of training have been shown to be equally effective in improving exercise capacity and lipid profile [107]. We did observe some potential confounding between session frequency and session time, with longer session times associated with less frequent exercise sessions. Despite this, however, session frequency alone was not predictive of MI outcomes, and the relationship between session time and myocardial infarction remained after adjusting for the frequency of sessions. Further investigation of the impact of session time on MI outcomes may therefore be warranted.

### Exercise Adherence and Mortality

Our finding of improved mortality outcomes with increased adherence to exercise training is supported by a similar observation in several cohort studies involving cardiac rehabilitation program attendees. Even when matched for prognostic factors, users of more cardiac rehabilitation sessions (≥70%) were less likely to die over the subsequent 5 years than lower users [124], and program completers had a reduced risk of death and hospitalisation than non-completers [125]. Additionally, Hammill [126] observed what appeared to be a dose-response gradient between sessions attended and the risk of myocardial infarction and mortality. Unfortunately, all these studies may have been subject to confounding if sicker participants were those who stopped attending earlier. Additionally, they were all based on adherence to the standardised US model of 36 supervised on-site sessions, meaning that outcomes observed may not necessarily be due to adherence alone, but the extra volume of training received. Given however that the results we observed in our study arose from adherence to programs of varying lengths and types and we found no effect of other training characteristics on mortality, adherence alone does appear to be an important factor.

While exercise adherence in itself may be an important driver behind reduced mortality, our findings could also be attributed to a phenomenon known as the ‘healthy adherer effect’ [127]. That is, those participants who complete more exercise sessions are also those who are more likely to adhere to medication regimens and perform other healthy lifestyle changes. Consequently, good exercise adherence could be considered a marker for adherence to other positive health behaviours which may also decrease the risk of mortality and clinical events. This has been previously observed by others, with increased attendance at on-site cardiac rehabilitation sessions related not only to reductions in all-cause mortality, re-admissions and major events, but also to greater improvements in clinical risk profile and secondary prevention behaviours such as medication and dietary adherence [128, 129]. Additionally, some ‘clustering’ of good lifestyle behaviours (diet, exercise, smoking cessation) was observed in survivors of MI, where increased adherence led to a decrease in mortality, myocardial infarction and stroke [130]. It could also be postulated however that cardiac rehabilitation programs in themselves promote adherence, by providing a structured learning environment which offers the frequent contact, skills training and self-efficacy enhancing care required for successful adherence [131]. Furthermore, knowledge about personal risk factors (usually provided at CR sessions) has been shown to correlate to a patient’s ability to achieve treatment goals [132]. Consequently, attending an increasing number of sessions provides increased chances to gain these skills and knowledge. Despite these difficulties in separating out the effect of exercise adherence, it does appear to play a crucial role in clinical outcomes, which has important implications for program design.

### Implications for Practice

There is a growing concern that cardiac rehabilitation needs to be re-engineered for the future, with a decreased reliance on traditional models of care [29, 133]. In order to do this, it is essential to understand the interaction between program components and patient outcomes to ensure essential elements are retained. In terms of exercise prescription at least, our findings appear to align well with the current impetus to provide flexible, individualised and ‘menu-based’ models of care tailored to circumstances and individual patient needs [134, 135]. While a number of the exercise training programs contained within our meta-regression were those of the traditional hospital-supervised, moderate-to-vigorous intensity format, many alternate styles were also observed. All appeared equally effective in reducing mortality, and most also in reducing the risk of subsequent myocardial infarction. It may be more important therefore to design exercise programs in whichever format that may help to promote increase participant adherence, rather than being only concerned with the details of specific intervention components.

By considering alternative evidence-based models of exercise training, and offering flexible program design in order to increase ongoing adherence to exercise, programs may also attract an increased number of participants to their service. While a lack of referral is often cited as a significant barrier to the uptake of cardiac rehabilitation, other well-known barriers include issues with the program itself such as exercise training location, session availability or the frequency and duration of attendance required [133, 136]. Knowledge that program design can be flexible, without greatly impacting on the clinical outcomes expected, may help to reassure clinicians when prescribing exercise training. Additionally, flexibility in design may overcome the resourcing issues faced by many programs in the current economic climate [137].

### Future Questions

It appears obvious that structured exercise training has clear benefits over usual care, even when this includes physical activity advice, yet the question remains as to the minimal threshold of exercise intensity and volume to produce these changes. This is a pertinent fact to consider given the rise of health coaching interventions in recent years which provide exercise interventions in a format somewhere between general advice and structured exercise intervention [138, 139]. Additionally, this review appears to support the hypothesis that the threshold for global health benefits may occur at low levels of intensity in people who are deconditioned. The minimum intensities in some included trials were lower than the aerobic intensity threshold for fitness improvements of 45% VO_2_R/60%HR_max_ proposed by Swain and Franklin [140], and consequently, it remains unclear at what point significant improvements in clinical outcome fail to occur.

### Strengths and Limitations

Our study is strengthened by the use of a broad search strategy with no limitations imposed on language or date of publication. We also performed sensitivity analysis to examine the effects of missing data on all outcomes. Additionally, by using an exhaustive process to contact trial authors for missing intervention details, we were able to provide a more complete picture of exercise training than has ever been previously examined. Our analysis contains a wide range of interventions, allowing a comparison of varying exercise characteristics across a number of clinical outcomes.

The strength of our findings however is limited by the quality of the included trials. Due to poor reporting, many were rated at unclear risk of bias, and missing data were observed in a number of trials. Importantly, however, imputation of these missing data in sensitivity analysis did not significantly change the effect of cardiac rehabilitation on clinical outcomes. Many intervention details also remained missing, limiting the amount of data available to perform meta-regression. In particular, attempts at multivariable analysis (to account for any potential confounding due to correlated co-variates) were limited by the low number of interventions reporting all exercise co-variates of interest. It is not clear if these missing variables would have changed the outcome of the analysis.

It must also be recognised that the results of subgroup analyses and meta-regression performed in our study are subject to the challenges associated with interpreting complex interventions in such a manner. Separating the effects of all potential effect modifiers is difficult, particularly where this information may be unreported or unaccounted for. It must also be considered that these component parts alone may not be enough to explain the power of an intervention. A complex intervention such as cardiac rehabilitation may be more than the sum of its parts, and in reducing it down into these parts, we may lose the essence of what makes it a successful system [141]. The outcomes of such interventions may also be context-dependant, and it is not yet clear how this can be accounted for in analyses [142].

Finally, while this review contains a large number of trials from many countries, two thirds of them are more than 15 years old, and the majority of participants are still middle-aged men. Therefore, the generalisability of these findings to modern cardiac rehabilitation practice including participants of more varied aetiologies, elderly patients and women may be somewhat compromised.

## Conclusions

This meta-analysis has shown that there is a place for exercise-based cardiac rehabilitation in the modern treatment of coronary heart disease, particularly where it plays a role in increasing the use of other secondary prevention therapies, many of which are currently suboptimal. There is little differential effect of variations in individual exercise training components, particularly on mortality outcomes. For this reason, it may be more important to offer programs which focus on achieving increased adherence to the exercise intervention, regardless of what format it may take.

## Additional file

Additional file 1: Supplementary Material 1. (DOCX 131 kb)
