# Supplementary material for: The Contribution of Individual Exercise Training Components to Clinical Outcomes in Randomised Controlled Trials of Cardiac Rehabilitation: A Systematic Review and Meta-regression
Source: Sports Med Open. 2017 May 5;3:19. doi: 10.1186/s40798-017-0086-z (PMC5419959; doi:10.1186/s40798-017-0086-z)
Supplement: Supplementary file 1 — Supplementary Material 1. (DOCX 131 kb) [file 40798_2017_86_MOESM1_ESM.docx]

Electronic Supplementary material for *“The contribution of individual exercise training components to clinical outcomes in randomised controlled trials of cardiac rehabilitation: a systematic review and meta-regression”*

Sports Medicine Open

Bridget Abell^a^, Paul Glasziou and Tammy Hoffmann

1. [babell@bond.edu.au](mailto:babell@bond.edu.au), Centre for Research in Evidence-Based Practice, Faculty of Health Sciences and Medicine, Bond University, Queensland, Australia

ESM Appendix S1. Database search strategies

**PubMed (via PubMed interface)**

1. ischemic heart disease [MeSH]

2. myocardial ischemia [MeSH]

3. coronary artery bypass [MeSH]

4. myocard* AND ischaemia[Text Word]

5. myocard* AND ischemia[Text Word]

6. ischaemi* AND heart[Text Word]

7. ischemi* AND heart[Text Word]

8. myocard* AND infarct*[Text Word]

9. heart infarct*[Text Word]

10. angina[Text Word]

11. PTCA[Text Word]

12. coronary disease*[Text Word]

13. coronary bypass[Text Word]

14. coronary thrombo*[Text Word]

15. coronary angioplast*[Text Word]

16. Exercise Therapy [MeSH]

17. Sports [MeSH:noexp]

18. Physical Exertion [MeSH:noexp]

19. rehabilitat*[Text Word]

20. physical* AND fit*[Text Word]

21. physical* AND train*[Text Word]

22. physical* AND therap*[Text Word]

23. physical* AND activit*[Text Word]

24. Exercise [MeSH]

25. train* AND strength*[Text Word]

26. train* AND aerobic*[Text Word]

27. train* AND exercise*[Text Word]

28. exercise* AND treatment[Text Word]

29. exercise* AND intervent*[Text Word]

30. exercise* AND program*

31. fitness treatment[Text Word]

32. fitness intervent*[Text Word]

33. fitness program*[Text Word]

34. Rehabilitation [MeSH]

35. kinesiotherap*[Text Word]

36. "Physical Education and Training" [MeSH]

37. aerobic* AND exercise*[Text Word]

38. exercise therap*[Text Word]

39. physical therapy [MeSH]

40. physiotherapy*[Text Word]

41. 1 OR 2 OR 3 OR 4 OR 5 OR 6 OR 7 OR 8 OR 9 OR 10 OR 11 OR 12 OR 13 OR 14 OR 15

42. 16 OR 17 OR 18 OR 19 OR 20 OR 21 OR 22 OR 23 OR 24 OR 24 OR 26 OR 27 OR 28 OR 29 OR 30 OR 31 OR 32 OR 33 OR 34 OR 35 OR 36 OR 37 OR 38 OR 39 OR 40

43. 41 AND 42

Filters activated: Randomized Controlled Trial, Systematic Reviews, Clinical Trial, Controlled Clinical Trial, Meta-Analysis, Humans

ESM Table S2. Modified TIDieR checklist^a^

| Item Name | Item Description |
| --- | --- |
| What: Procedures | Describes each of the procedures, activities, and/or processes used in the intervention, including any enabling or support activities e.g. mode of exercise |
| How | Describes the modes of delivery (e.g. face-to-face, internet) of the intervention and whether it was provided individually or in a group |
| Where | Describes the type(s) of location(s) where the intervention occurred, including any necessary infrastructure or relevant features |
| Provider | Describes the intervention provider and their expertise, background, and any specific training given |
| When and How Much   1. *Intensity* 2. *Frequency* 3. *Session Time* 4. *Overall Duration* | Describes the dose/schedule of the intervention including:  *The intensity of exercise used in the intervention (e.g. % heart rate)*  *The frequency of exercise sessions*  *The duration of each individual exercise session*  *The overall duration of the exercise intervention* |
| How Well: Actual | Describes the extent to which the intervention was delivered as planned (if adherence or fidelity was assessed) |

1. Adapted from Hoffmann TC, Glasziou PP, Boutron I, et al. Better reporting of interventions: template for intervention description and replication (TIDieR) checklist and guide. BMJ. 2014;348:g1687

ESM Appendix S3. Details about abstracts excluded from review

**Abstracts with no response from authors**

1. Ghroubi S, Elleuch W, Abid L, Kammoun S, Elleuch MH. The effects of cardiovascular rehabilitation after coronary stenting. Ann Phys Rehabil Med. 2012;55:S1 e309. *Presented at 27e Congrès de Médecine Physique et de Réadaptation, 2012.*

**Abstracts with outcomes eligible for inclusion in review but author failed to respond to further requests for data**

1. Vona M, Iannino T, De Pascalis S, Gallardo C, Cook S, Vermeulen B. Impact of Endothelial Dysfunction Improvement After Exercise Training on Prognosis in Patients With Recent Myocardial Infarction. Circulation. 2010;122(Suppl 21):A13764.  *Presented at American Heart Association Scientific Sessions, 2010.*

**Abstracts with outcomes eligible for inclusion in review but authors not yet ready for data to be published**

1. Hadadzadeh MH, Maiya AG, Shad B, Mirbolouk F, PadmaKumar R, Borkar SS, Devasia T, Raman VG, Nair S, Guddattu V. Abstract P280: Home versus Hospital-Based Exercise Training and Associated Improvements in Functional Capacity and Quality of Life in Post-event Coronary Artery Disease Patients: An Indo-Iranian Multi-center Randomized Controlled Trial. Circulation. 2015;131(Suppl 1):AP280. *Presented at EPI/Lifestyle*2015 *conference.*
2. Vataman EB, Grivenco, AA, Filimon SS, Lisii DM, Morcov, LG. Effect of long-term home based cardiac rehabilitation programme on readmission rate and mortality risk after coronary revascularisation. *Abstract P3659 at the European Society of Cardiology Congress 2015.*

ESM Figures S4. Funnel plots for publication bias


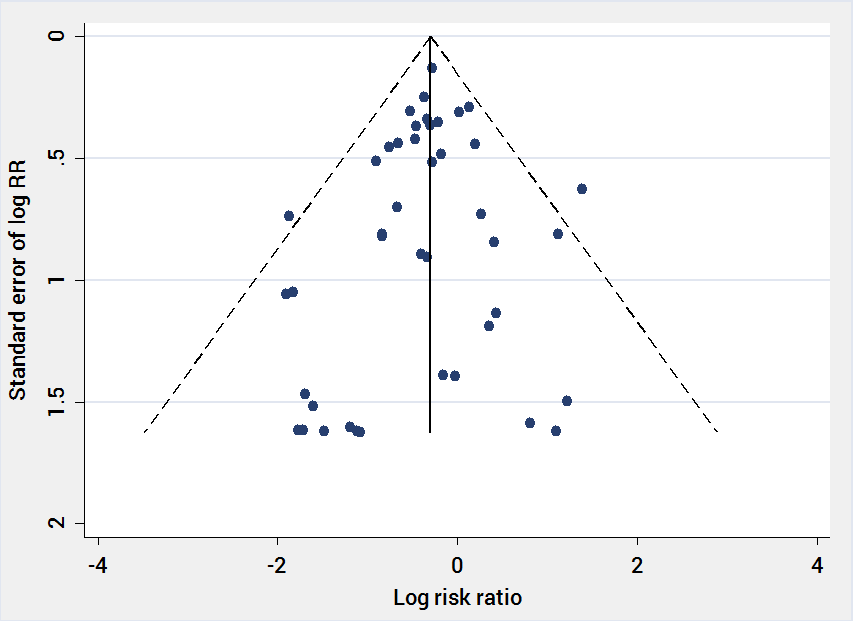


**Fig S4a**. Funnel plot with pseudo 95% confidence limits: cardiovascular mortality


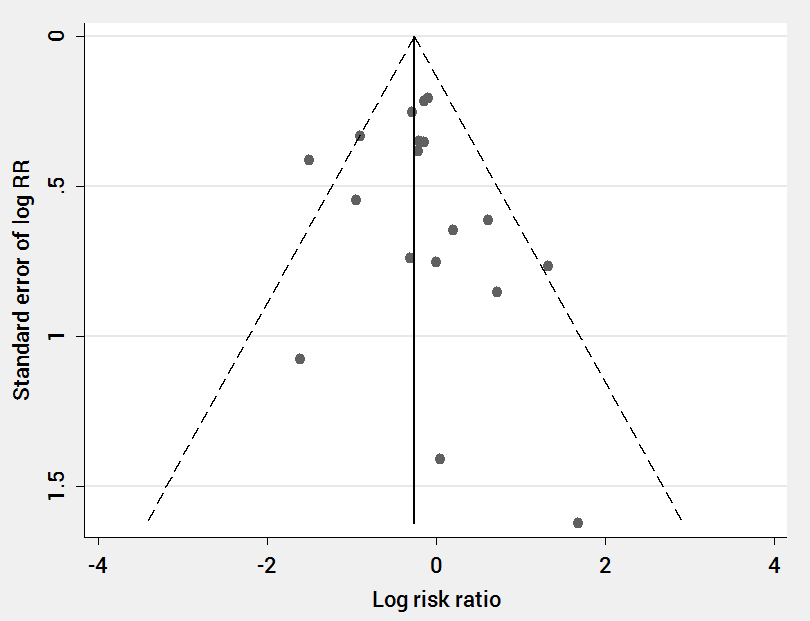


**Fig S4b**. Funnel plot with pseudo 95% confidence limits: percutaneous coronary intervention

ESM Appendix S5. Risk of Bias assessment

**Table S5**. Risk of Bias assessment for individual trials

| **Trial, Year** | **Assessment Criteria** | | | |
| --- | --- | --- | --- | --- |
|  | Random sequence generation | Allocation concealment | Blinded or objective outcomes | Missing outcome data |
| Albus, 2009 | Low risk | Low risk | Low risk | Low risk |
| Andersen, 1981 | Low risk | Unclear | Low risk | Low risk |
| Aronov, 2009 | Unclear | Unclear | Low risk | Low risk |
| Belardinelli, 2001/07 | Unclear | Unclear | Unclear | Unclear |
| Bell, 1998 | Unclear | Low risk | Low risk | Low risk |
| Bengtsson, 1983 | Unclear | Unclear | Unclear | High risk |
| Bertie, 1992 | Unclear | Unclear | Unclear | High risk |
| Bethell, 1990/99 | Low risk | Unclear | Low risk | Low risk |
| Blumenthal, 2005 | Low risk | Low risk | Unclear | Low risk |
| Briffa, 2005 | Low risk | Low risk | Low risk | Low risk |
| Byrkjeland, 2015 | Low risk | Low risk | Unclear | Low risk |
| Carlsson, 1998 | Unclear | Unclear | Unclear | Low risk |
| Carson, 1982 | Unclear | Unclear | Unclear | Low risk |
| DeBusk, 1994 | Low risk | Low risk | Low risk | Low risk |
| Dugmore, 1999 | Unclear | Unclear | High risk | Low risk |
| Engblom, 1992/97 | Unclear | Unclear | Unclear | Low risk |
| Erdman, 1986 | Low risk | Unclear | Unclear | High risk |
| Ferreira, 2010 | Low risk | Unclear | Low risk | Low risk |
| Fletcher, 1994 | Unclear | Unclear | Unclear | Low risk |
| Fontes-Carvalho, 2015 | Low risk | Unclear | Unclear | Low risk |
| Fridlund, 1992/Lidell, 1996 | Unclear | Unclear | Low risk | High risk |
| Giallauria, 2008 | Unclear | Unclear | Unclear | Low risk |
| Haglin, 2011 | High risk | Unclear | Unclear | Low risk |
| Haskell, 1994 | Low risk | Low risk | High risk | Low risk |
| Hofman-Bang, 1995/Lisspers 2005 | Unclear | Unclear | Low risk | Low risk |
| Holmbäck, 1994 | Low risk | Low risk | Unclear | Low risk |
| Kallio, 1979/ Hämäläinen, 1995 | Low risk | Unclear | Low risk | Low risk |
| Kovoor, 2006 | Unclear | Low risk | Unclear | High risk |
| Krasnitskiĭ, 2010 | Unclear | Unclear | Low risk | Low risk |
| La Rovere, 2002 | Unclear | Unclear | Low risk | Low risk |
| Lear, 2014 | Low risk | Low risk | Low risk | Low risk |
| Leizorovicz, 1991 (PRECOR) | Unclear | Unclear | Low risk | Low risk |
| Marchionni, 2003 | Unclear | Unclear | Low risk | Low risk |
| Miller, 1984 | Unclear | Unclear | Unclear | Low risk |
| **Trial, Year** | **Assessment Criteria** | | | |
|  | Random sequence generation | Allocation concealment | Blinded or objective outcomes | Missing outcome data |
| Maroto Montero, 1996/2005 | Unclear | Unclear | Low risk | Low risk |
| Munk, 2009 | Low risk | Low risk | Low risk | Low risk |
| Mutwalli, 2012 | Unclear | Unclear | Low risk | Low risk |
| Oerkild, 2012 | Low risk | Low risk | Low risk | Low risk |
| Oldridge, 1991 | Unclear | Unclear | High risk | Low risk |
| Ornish, 1990/1998 | Unclear | Unclear | Low risk | High risk |
| Reid, 2012 | Low risk | Low risk | Low risk | High risk |
| Román, 1983 | Unclear | Unclear | Unclear | High risk |
| Schuler, 1992/Niebauer, 1997 | Unclear | Low risk | Unclear | Low risk |
| Shaw, 1981/Dorn, 1999 | Unclear | Unclear | Low risk | Low risk |
| Sivarajan, 1982 | Unclear | Unclear | Low risk | Low risk |
| Specchia, 1996 | Unclear | Unclear | Unclear | Low risk |
| Ståhle, 1999/Hage, 2003 | Unclear | Unclear | Unclear | Low risk |
| Stern, 1983 | Unclear | Unclear | Unclear | Low risk |
| Toobert, 2000 | Unclear | Unclear | Unclear | Low risk |
| Vecchio, 1981 | Low risk | Unclear | Unclear | High risk |
| Vermeulen, 1983 | Unclear | Unclear | Unclear | Low risk |
| Vestfold Heart Care Group, 2003 | Unclear | Low risk | Low risk | Low risk |
| Wang, 2012 | Low risk | Unclear | Low risk | Low risk |
| West, 2013 | Low risk | Low risk | Low risk | Low risk |
| WHO Balatonfured, 1983 | Low risk | Unclear | Unclear | Low risk |
| WHO Brussels, 1983 | Low risk | Unclear | Unclear | Low risk |
| WHO Bucharest, 1983 | Low risk | Unclear | Unclear | Low and High risk^a^ |
| WHO Budapest, 1983 | Low risk | Unclear | Unclear | Low risk |
| WHO Dessau, 1983 | Low risk | Unclear | Unclear | Low risk |
| WHO Erfurt, 1983 | Low risk | Unclear | Unclear | Low risk |
| WHO Ghent, 1983 | Low risk | Unclear | Unclear | Low risk |
| WHO Kaunas, 1983 | Low risk | Unclear | Unclear | Low risk |
| WHO Prauge, 1983 | Low risk | Unclear | Unclear | Low risk |
| WHO Rome, 1983 | Low risk | Unclear | Unclear | Low and High risk^a^ |
| WHO Tel Aviv, 1983 | Low risk | Unclear | Unclear | Low risk |
| WHO Warsaw, 1983 | Low risk | Unclear | Unclear | High risk |
| Wilhelmsen, 1975 | Low risk | Unclear | Low risk | Low risk |
| Yu, 2004 | Unclear | Unclear | Low risk | High risk |
| Zwistler, 2008 | Low risk | Low risk | Low risk | Low risk |

^a^rated low risk of bias for mortality outcomes but high risk of bias for myocardial infarction, CABG and PCI outcome data

**Fig S5a**. Risk of bias graph for all included studies reporting mortality outcomes (n=65)

**Fig S5b**. Risk of bias graph for all included studies reporting myocardial infarction, CABG and PCI outcomes (n=60)

ESM Table S6. Sensitivity analysis: comparison of available case analysis with effect estimates obtained via imputation of missing participant data or exclusion of trials at high risk of bias for missing outcome data

Method 1: event rate in all missing participants equal to that of those followed up in trial control arm.

Method 2: event rate in participants missing from trial intervention arm 1.5 times those followed up in same arm; event rate in participants missing from trial control arm the same as those followed up in the same arm.

Method 3: excluding studies rated at high risk of bias for missing outcome data

| S6a. Cardiovascular Mortality | Relative Risk | 95% CI | p-value | I^2^ |
| --- | --- | --- | --- | --- |
| Available case analysis | 0.74 | 0.65-0.86 | <0.0001 | 0% |
| Imputation of missing data (Method 1) | 0.75 | 0.66-0.86 | <0.0001 | 0% |
| Imputation of missing data (Method 2) | 0.76 | 0.66-0.88 | 0.0001 | 1% |
| Exclude studies at high risk of bias (Method 3) | 0.75 | 0.65-0.87 | 0.0001 | 0% |

| S6b. Total Mortality | Relative Risk | 95% CI | p-value | I^2^ |
| --- | --- | --- | --- | --- |
| Available case analysis | 0.90 | 0.83-0.99 | 0.03 | 0% |
| Imputation of missing data (Method 1) | 0.90 | 0.82-0.98 | 0.02 | 0% |
| Imputation of missing data (Method 2) | 0.91 | 0.84-1.00 | 0.04 | 0% |
| Exclude studies at high risk of bias (Method 3) | 0.92 | 0.84-1.01 | 0.08 | 0% |

| S6c. Myocardial Infarction | Relative Risk | 95% CI | p-value | I^2^ |
| --- | --- | --- | --- | --- |
| Available case analysis | 0.80 | 0.70-0.92 | 0.002 | 0% |
| Imputation of missing data (Method 1) | 0.83 | 0.73-0.94 | 0.004 | 0% |
| Imputation of missing data (Method 2) | 0.83 | 0.73-0.95 | 0.005 | 0% |
| Exclude studies at high risk of bias (Method 3) | 0.83 | 0.72-0.96 | 0.01 | 0% |

| S6d. Coronary Artery Bypass Grafting | Relative Risk | 95% CI | p-value | I^2^ |
| --- | --- | --- | --- | --- |
| Available case analysis | 0.96 | 0.79-1.15 | 0.64 | 0% |
| Imputation of missing data (Method 1) | 0.94 | 0.79-1.13 | 0.88 | 0% |
| Imputation of missing data (Method 2) | 0.95 | 0.79-1.13 | 0.74 | 0% |
| Exclude studies at high risk of bias (Method 3) | 1.01 | 0.83-1.23 | 0.89 | 0% |

| S6e. Percutaneous Coronary Intervention | Relative Risk | 95% CI | p-value | I^2^ |
| --- | --- | --- | --- | --- |
| Available case analysis | 0.77 | 0.59-1.00 | 0.05 | 37% |
| Imputation of missing data (Method 1) | 0.80 | 0.64-1.00 | 0.05 | 28% |
| Imputation of missing data (Method 2) | 0.81 | 0.62-1.06 | 0.12 | 45% |
| Exclude studies at high risk of bias (Method 3) | 0.78 | 0.59-1.02 | 0.07 | 32% |

ESM Table S7. Sensitivity analysis: comparison of available case analysis with effect estimates obtained after excluding data from trials only available in abstract or doctoral form

| S7a. Cardiovascular Mortality | Interventions  (n) | Relative Risk | 95% CI | p-value | I^2^ |
| --- | --- | --- | --- | --- | --- |
| Available case analysis | 44 | 0.74 | 0.65-0.86 | <0.0001 | 0% |
| Excluding abstract/doctoral data | 43 | 0.76 | 0.66-0.87 | 0.0001 | 0% |

| S7b. Total Mortality | Interventions  (n) | Relative Risk | 95% CI | p-value | I^2^ |
| --- | --- | --- | --- | --- | --- |
| Available case analysis | 60 | 0.90 | 0.83-0.99 | 0.03 | 0% |
| Excluding abstract/doctoral data | 57 | 0.90 | 0.83-0.99 | 0.03 | 0% |

| S7c. Myocardial Infarction | Interventions  (n) | Relative Risk | 95% CI | p-value | I^2^ |
| --- | --- | --- | --- | --- | --- |
| Available case analysis | 53 | 0.80 | 0.70-0.92 | 0.002 | 0% |
| Excluding abstract/doctoral data | 52 | 0.81 | 0.70-0.92 | 0.002 | 0% |

| S7d. Percutaneous Coronary Intervention | Interventions  (n) | Relative Risk | 95% CI | p-value | I^2^ |
| --- | --- | --- | --- | --- | --- |
| Available case analysis | 18 | 0.77 | 0.59-1.00 | 0.05 | 37% |
| Exclude abstract/doctoral data | 18 | 0.81 | 0.65-1.00 | 0.05 | 13% |

***S7e. Coronary Artery Bypass Grafting:*** no abstract or doctoral data included

ESM Tables S8. Subgroup analysis of the differing effects of intervention and trial characteristics on outcomes.

**Table S8a.** Cardiovascular mortality outcome: subgroup analysis

| **Trial or intervention characteristic** | **Number of interventions with this characteristic** | **Relative Risk of outcome (95% CI)** | **I^2^** | **p-value** | **Interaction effect across subgroups** |
| --- | --- | --- | --- | --- | --- |
| Population diagnosis |  |  |  |  | Not significant (p=0.60, I^2^=0%) |
| Myocardial Infarction only | 31 | 0.75 (0.65-0.87) | 0% | 0.0001 |  |
| Mixed or other aetiologies | 13 | 0.63 (0.33-1.19) | 10% | 0.16 |  |
| Amount of exercise prescribed per week |  |  |  |  | Not significant (p=0.81, I^2^=0%) |
| ≥ 150 minutes | 25 | 0.72 (0.56-0.92) | 11% | 0.009 |  |
| < 150 minutes | 7 | 0.75 (0.55-1.03) | 0% | 0.07 |  |
| Type of control arm |  |  |  |  | Not significant (p=0.72, I^2^=0%) |
| Usual care | 28 | 0.76 (0.62-0.94) | 0% | 0.01 |  |
| Usual care plus lifestyle advice | 16 | 0.72 (0.56-0.92) | 13% | 0.008 |  |
| Lipid lowering therapy |  |  |  |  |  |
| Not present in any trial arm | 31 | 0.73 (0.63-0.85) | 0% | <0.0001 | Not significant (p=0.60, I^2^=0%) |
| Used in all trial arms | 11 | 0.76 (0.37-1.55) | 0% | 0.45 |  |
| Used only in intervention arm | 2 | 1.10 (0.51-2.40) | 0% | 0.81 |  |

**Table S8b**. Total mortality outcome: subgroup analysis

| **Trial or intervention characteristic** | **Number of interventions with this characteristic** | **Relative Risk of outcome (95% CI)** | **I^2^** | **p-value** | **Interaction effect across subgroups** |
| --- | --- | --- | --- | --- | --- |
| Population diagnosis |  |  |  |  | Not significant (p=0.95, I^2^=0%) |
| Myocardial Infarction only | 38 | 0.90 (0.82-0.99) | 0% | 0.03 |  |
| Mixed or other aetiologies | 22 | 0.91 (0.67-1.24) | 0% | 0.55 |  |
| Amount of exercise prescribed per week |  |  |  |  | Not significant (p=0.49, I^2^=0%) |
| ≥ 150 minutes | 27 | 0.88 (0.75-1.04) | 0% | 0.12 |  |
| < 150 minutes | 18 | 0.95 (0.84-1.07) | 0% | 0.37 |  |
| Type of control arm |  |  |  |  | Not significant (p=0.33, I^2^=0%) |
| Usual care | 30 | 0.84 (0.71-1.00) | 0% | 0.05 |  |
| Usual care plus lifestyle advice | 30 | 0.93 (0.84-1.03) | 0% | 0.18 |  |
| Lipid lowering therapy |  |  |  |  |  |
| Not present in any trial arm | 34 | 0.83 (0.73-0.94) | 0% | 0.004 | Some difference in effect based on presence of lipid lowering therapy observed (I^2^=47.6%) but does not reach significance (p=0.15). |
| Used in all trial arms | 23 | 0.98 (0.86-1.12) | 0% | 0.82 |  |
| Used only in intervention arm | 3 | 1.15 (0.58-2.27) | 0% | 0.69 |  |

**Table S8c**. Myocardial infarction outcome: subgroup analysis

| **Trial or intervention characteristic** | **Number of interventions with this characteristic** | **Relative Risk of outcome (95% CI)** | **I^2^** | **p-value** | **Interaction effect across subgroups** |
| --- | --- | --- | --- | --- | --- |
| Population diagnosis |  |  |  |  | Not significant (p=0.76, I^2^=0%) |
| Myocardial Infarction only | 38 | 0.81 (0.70-0.94) | 2% | 0.005 |  |
| Mixed or other aetiologies | 15 | 0.75 (0.49-1.16) | 0% | 0.19 |  |
| Amount of exercise prescribed per week |  |  |  |  | Not significant (p=0.89, I^2^=0%) |
| ≥ 150 minutes | 26 | 0.82 (0.63-1.06) | 9% | 0.13 |  |
| < 150 minutes | 14 | 0.80 (0.64-0.99) | 0% | 0.04 |  |
| Type of control arm |  |  |  |  | Not significant (p=0.96, I^2^=0%) |
| Usual care | 31 | 0.79 (0.66-0.96) | 0% | 0.02 |  |
| Usual care plus lifestyle advice | 22 | 0.80 (0.64-1.00) | 6% | 0.05 |  |
| Lipid lowering therapy |  |  |  |  |  |
| Not present in any trial arm | 34 | 0.82 (0.70-0.95) | 0% | 0.01 | Some difference in effect based on presence of lipid lowering therapy observed (I^2^=32.5%) but does not reach significance (p=0.23). Driven by final subgroup where LLT used as part of intervention. |
| Used in all trial arms | 17 | 0.87 (0.63-1.20) | 0% | 0.40 |  |
| Used only in intervention arm | 2 | 0.48 (0.26-0.89) | 0% | 0.02 |  |

**Table S8d.** CABG outcome: subgroup analysis

| **Trial or intervention characteristic** | **Number of interventions with this characteristic** | **Relative Risk of outcome (95% CI)** | **I^2^** | **p-value** | **Interaction effect across subgroups** |
| --- | --- | --- | --- | --- | --- |
| Population diagnosis |  |  |  |  | Significant difference in effect based on the diagnosis of the population included in the intervention. (p=0.04, I^2^=76%) |
| Myocardial Infarction only | 17 | 1.08 (0.89-1.34) | 0% | 0.51 |  |
| Mixed or other aetiologies | 14 | 0.69 (0.48-0.99) | 0% | 0.05 |  |
| Amount of exercise prescribed per week |  |  |  |  | Not significant (p=0.98, I^2^=0%) |
| ≥ 150 minutes | 22 | 0.97 (0.78-1.21) | 0% | 0.78 |  |
| < 150 minutes | 6 | 0.98 (0.67-1.42) | 0% | 0.90 |  |
| Type of control arm |  |  |  |  | Not significant (p=0.45, I^2^=0%) |
| Usual care | 11 | 0.84 (0.58-1.23) | 0% | 0.38 |  |
| Usual care plus lifestyle advice | 20 | 1.00 (0.80-1.24) | 0% | 0.98 |  |
| Lipid lowering therapy |  |  |  |  |  |
| Not present in any trial arm | 17 | 0.95 (0.69-1.32) | 0% | 0.78 | Not significant (p=0.95, I^2^=0%) |
| Used in all trial arms | 12 | 0.90 (0.67-1.20) | 0% | 0.48 |  |
| Used only in intervention arm | 2 | 0.83 (0.31-2.24) | 74% | 0.72 |  |

**Table S8e**. PCI outcome: subgroup analysis

| **Trial or intervention characteristic** | **Number of interventions with this characteristic** | **Relative Risk of outcome (95% CI)** | **I^2^** | **p-value** | **Interaction effect across subgroups** |
| --- | --- | --- | --- | --- | --- |
| Population diagnosis |  |  |  |  | Not significant (p=0.45, I^2^=0%) |
| Myocardial Infarction only | 5 | 0.87 (0.64-1.18) | 0% | 0.37 |  |
| Mixed or other aetiologies | 13 | 0.72 (0.50-1.05) | 51% | 0.08 |  |
| Amount of exercise prescribed per week |  |  |  |  | Not significant (p=0.96, I^2^=0%) |
| ≥ 150 minutes | 14 | 0.79 (0.55-1.15) | 48% | 0.23 |  |
| < 150 minutes | 3 | 0.80 (0.57-1.13) | 0% | 0.21 |  |
| Type of control arm |  |  |  |  | Not significant (p=0.87, I^2^=0%) |
| Usual care | 7 | 0.80 (0.60-1.07) | 0% | 0.13 |  |
| Usual care plus lifestyle advice | 11 | 0.84 (0.55-1.27) | 56% | 0.40 |  |
| Lipid lowering therapy |  |  |  |  |  |
| Not present in any trial arm | 4 | 0.46 (0.20-1.06) | 52% | 0.07 | Some difference in effect based on the presence of lipid lowering therapy observed (I^2^=21.1%) but does not reach significance (p=0.28). |
| Used in all trial arms | 12 | 0.90 (0.72-1.13) | 0% | 0.38 |  |
| Used only in intervention arm | 2 | 0.78 (0.52-1.16) | 0% | 0.22 |  |

ESM Tables S9. Univariate regression exploring the impact of intervention co-variates on coronary heart disease outcomes

**Table S9a.** Univariate regression: cardiovascular mortality

| **Intervention characteristic/co-variate, number reporting** | **Co-efficient of model**^a^ **(95% Confidence Interval)** | **p-value** | **Adjusted R^2^** |
| --- | --- | --- | --- |
| *Continuous co-variates* |  |  |  |
| Median year of participant recruitment, n=30 | 0.99 (0.97-1.02) | 0.434 | n/a |
| Total intervention duration (months), n=41 | 1.00 (0.99-1.01) | 0.912 | n/a |
| Exercise session frequency (times/week), n=33 | 0.95 (0.83-1.09) | 0.476 | n/a |
| Exercise session time (minutes), n=32 | 1.00 (0.99-1.01) | 0.524 | n/a |
| Exercise intensity: lowest prescribed (%HRmax), n=29 | 0.99 (0.97-1.01) | 0.391 | n/a |
| Exercise intensity: highest prescribed (%HRmax), n=29 | 1.00 (0.98-1.02) | 0.881 | n/a |
|  |  |  |  |
| *Categorical co-variates* |  |  |  |
| Mode of exercise training, n=31 |  |  |  |
| Aerobic and resistance training (vs. aerobic only) | 0.88 (0.56-1.39) | 0.572 | n/a |
| How exercise training was delivered, n=37 |  |  |  |
| Group-based sessions (vs. training individually) | 0.95 (0.61-1.48) | 0.814 | n/a |
| Exercise provider, n=23 |  |  |  |
| Included a physician in team (vs. no physician) | 0.97 (0.64-1.46) | 0.869 | n/a |
| Adherence to intervention, n=32 |  |  |  |
| High (vs. moderate) | 0.72 (0.52-0.99) | 0.045^bc^ | d |

a, expressed as rate of change in relative risk with increasing levels of the co-variate e.g. an increased relative risk means that the characteristic increases the relative risk of the outcome as it also increases; b, met criteria for entry in multi-variate regression model; c, adjusted p-value remained <0.05 after accounting for multiple hypothesis testing (using permute 5000); d, could not be calculated as no residual heterogeneity observed in original model; R^2^, proportion of heterogeneity/variability in outcome explained by co-variate

**Table S9b.** Univariate regression: Total mortality

| **Intervention characteristic/co-variate, number reporting** | **Co-efficient of model**^a^ **(95% Confidence Interval)** | **p-value** | **Adjusted R^2^** |
| --- | --- | --- | --- |
| *Continuous co-variates* |  |  |  |
| Median year of participant recruitment, n=44 | 1.01 (1.00-1.01) | 0.113 ^b^ | n/a |
| Total intervention duration (months), n=60 | 1.00 (0.99-1.00) | 0.198 ^b^ | n/a |
| Exercise session frequency (times/week), n=49 | 0.96 (0.90-1.03) | 0.253 | n/a |
| Exercise session time (minutes), n=45 | 1.00 (1.00-1.01) | 0.289 | n/a |
| Exercise intensity: lowest prescribed (%HRmax), n=36 | 0.99 (0.98-1.01) | 0.411 | n/a |
| Exercise intensity: highest prescribed (%HRmax), n=35 | 1.01 (0.99-1.03) | 0.272 | n/a |
|  |  |  |  |
| *Categorical co-variates* |  |  |  |
| Mode of exercise training, n=45 |  |  |  |
| Aerobic and resistance training (vs. aerobic only) | 1.06 (0.75-1.49) | 0.742 | n/a |
| How exercise training was delivered, n=58 |  |  |  |
| Group-based sessions (vs. training individually) | 1.05 (0.74-1.49) | 0.793 | n/a |
| Exercise provider, n=45 |  |  |  |
| Included a physician in team (vs. no physician) | 0.87 (0.70-1.10) | 0.235 | n/a |
| Adherence to intervention, n=39 |  |  |  |
| High (vs. moderate) | 0.81 (0.66-0.996) | 0.046^bc^ | 100% |

a, expressed as rate of change in relative risk with increasing levels of the co-variate e.g. an increased relative risk means that the characteristic increases the relative risk of the outcome as it also increases; b, met criteria for entry in multi-variate regression model; c, adjusted p-value 0.06 after accounting for multiple hypothesis testing (using permute 5000); R^2^, proportion of heterogeneity/variability in outcome explained by co-variate

**Table S9c**. Univariate regression: Myocardial Infarction

| **Intervention characteristic/co-variate, number reporting** | **Co-efficient of model**^a^ **(95% Confidence Interval)** | **p-value** | **Adjusted R^2^** |
| --- | --- | --- | --- |
| *Continuous co-variates* |  |  |  |
| Median year of participant recruitment, n=36 | 1.00 (0.98-1.01) | 0.620 | n/a |
| Total intervention duration (months), n=51 | 1.00 (0.99-1.00) | 0.230 | n/a |
| Exercise session frequency (times/week), n=43 | 0.90 (0.80-1.02) | 0.136^b^ | n/a |
| Exercise session time (minutes), n=41 | 1.01 (1.00-1.02) | 0.011 ^bc^ | 100% |
| Exercise intensity: lowest prescribed (%HRmax), n=35 | 1.01 (0.99-1.03) | 0.516 | n/a |
| Exercise intensity: highest prescribed (%HRmax), n=37 | 0.99 (0.97-1.02) | 0.450 | n/a |
|  |  |  |  |
| *Categorical co-variates* |  |  |  |
| Mode of exercise training, n=39 |  |  |  |
| Aerobic and resistance training (vs. aerobic only) | 1.39 (0.90-2.15) | 0.129 ^b^ | n/a |
| How exercise training was delivered, n=47 |  |  |  |
| Group-based sessions (vs. training individually) | 1.32 (0.89-1.96) | 0.169^b^ | n/a |
| Exercise provider, n=34 |  |  |  |
| Included a physician in team (vs. no physician) | 1.39 (0.92-2.11) | 0.113 ^b^ | n/a |
| Adherence to intervention, n=39 |  |  |  |
| High (vs. moderate) | 1.02 (0.74-1.41) | 0.886 | n/a |

a, expressed as rate of change in relative risk with increasing levels of the co-variate e.g. an increased relative risk means that the characteristic increases the relative risk of the outcome as it also increases; b, met criteria for entry in multi-variate regression model; c, adjusted p-value remained <0.05 after accounting for multiple hypothesis testing (using permute 5000); R^2^, proportion of heterogeneity/variability in outcome explained by co-variate

**Table S9d**. Univariate regression: CABG

| **Intervention characteristic/co-variate, number reporting** | **Co-efficient of model**^a^ **(95% Confidence Interval)** | **p-value** | **Adjusted R^2^** |
| --- | --- | --- | --- |
| *Continuous co-variates* |  |  |  |
| Median year of participant recruitment, n=19 | 1.00 (0.97-1.03) | 0.892 | n/a |
| Total intervention duration (months), n=29 | 0.99 (0.98-1.00) | 0.152^b^ | n/a |
| Exercise session frequency (times/week), n=29 | 1.00 (0.89-1.13) | 0.975 | n/a |
| Exercise session time (minutes), n=28 | 1.00 (0.99-1.01) | 0.572 | n/a |
| Exercise intensity: lowest prescribed (%HRmax), n=24 | 1.00 (0.98-1.03) | 0.952 | n/a |
| Exercise intensity: highest prescribed (%HRmax), n=24 | 1.02 (0.98-1.06) | 0.390 | n/a |
|  |  |  |  |
| *Categorical co-variates* |  |  |  |
| Mode of exercise training, n=26 |  |  |  |
| Aerobic and resistance training (vs. aerobic only) | 1.17 (0.69-1.97) | 0.539 | n/a |
| How exercise training was delivered, n=28 |  |  |  |
| Group-based sessions (vs. training individually) | 0.88 (0.54-1.44) | 0.603 | n/a |
| Exercise provider, n=23 |  |  |  |
| Included a physician in team (vs. no physician) | 0.99 (0.63-1.57) | 0.973 | n/a |
| Adherence to intervention, n=21 |  |  |  |
| High (vs. moderate) | 0.84 (0.48-1.40) | 0.475 | n/a |

a, expressed as rate of change in relative risk with increasing levels of the co-variate e.g. an increased relative risk means that the characteristic increases the relative risk of the outcome as it also increases; b, met criteria for entry in multi-variate regression model; R^2^, proportion of heterogeneity/variability in outcome explained by co-variate.

**Table S9e.** Univariate regression: PCI

| **Intervention characteristic/co-variate, number reporting** | **Co-efficient of model**^a^ **(95% Confidence Interval)** | **p-value** | **Adjusted R^2^** |
| --- | --- | --- | --- |
| *Continuous co-variates* |  |  |  |
| Median year of participant recruitment, n=11 | 1.02 (0.96-1.08) | 0.531 | n/a |
| Total intervention duration (months), n=18 | 1.00 (0.98-1.02) | 0.957 | n/a |
| Exercise session frequency (times/week), n=17 | 0.93 (0.74-1.15) | 0.456 | n/a |
| Exercise session time (minutes), n=17 | 1.01 (0.99-1.02) | 0.530 | n/a |
| Exercise intensity: lowest prescribed (%HRmax), n=15 | 1.02 (0.97-1.07) | 0.413 | n/a |
| Exercise intensity: highest prescribed (%HRmax), n=15 | 1.05 (1.00-1.11) | 0.047^bc^ | 72% |
|  |  |  |  |
| *Categorical co-variates* |  |  |  |
| Mode of exercise training, n=15 |  |  |  |
| Aerobic and resistance training (vs. aerobic only) | 1.23 (0.54-2.79) | 0.594 | n/a |
| How exercise training was delivered, n=17 |  |  |  |
| Group-based sessions (vs. training individually) | 0.98 (0.42-2.31) | 0.967 | n/a |
| Exercise provider, n=13 |  |  |  |
| Included a physician in team (vs. no physician) | 0.30 (0.14-0.62) | 0.004^b c^ | 100% |
| Adherence to intervention, n=13 |  |  |  |
| High (vs. moderate) | 0.63 (0.19-2.08) | 0.409 | n/a |

a, expressed as rate of change in relative risk with increasing levels of the co-variate e.g. an increased relative risk means that the characteristic increases the relative risk of the outcome as it also increases; b, met criteria for entry in multi-variate regression model; c, adjusted p-value remained <0.05 after accounting for multiple hypothesis testing (using permute 5000); R^2^, proportion of heterogeneity/variability in outcome explained by co-variate
